# Modeling with graded interfaces: Tool for understanding and designing record-high power and efficiency mid-infrared quantum cascade lasers

**DOI:** 10.1515/nanoph-2023-0687

**Published:** 2024-01-12

**Authors:** Suraj Suri, Benjamin B. Knipfer, Thomas Grange, Huilong Gao, Jeremy D. Kirch, Luke J. Mawst, Robert A. Marsland, Dan Botez

**Affiliations:** University of Wisconsin-Madison, Madison, WI, 53706, USA; Intraband, LLC, Madison, WI, 53726, USA; nextnano Lab, 12 chemin des prunelles, 38700 Corenc, France

**Keywords:** interface-roughness scattering, graded-interfaces superlattices, photon-induced carrier transport, carrier leakage in mid-infrared quantum cascade lasers, high wall-plug efficiency

## Abstract

By employing a graded-interfaces model based on a generalized formalism for interface-roughness (IFR) scattering that was modified for mid-infrared emitting quantum cascade lasers (QCLs), we have accurately reproduced the electro-optical characteristics of published record-performance 4.9 µm- and 8.3 µm-emitting QCLs. The IFR-scattering parameters at various interfaces were obtained from measured values and trends found via atom-probe tomography analysis of one of our 4.6 μm-emitting QCL structures with variable barrier heights. Those values and trends, when used for designing a graded-interface, 4.6 μm-emitting QCL, led to experimental device characteristics in very good agreement with calculated ones. We find that the published record-high performance values are mainly due to both injection from a prior-stage low-energy (active-region) state *directly* into the upper-laser (*ul*) level, thus at low field-strength values, as well as to strong photon-induced carrier transport. However, the normalized leakage-current density *J*_*leak*_/*J* is found to be quite high: 26–28 % and 23.3 %, respectively, mainly because of IFR-triggered shunt-type leakage through high-energy active-region states, in the presence of high average electron temperatures in the *ul* laser level and an energy state adjacent to it: 1060 K and 466 K for 4.9 µm- and 8.3 µm-emitting QCLs, respectively. Then, modeling with graded interfaces becomes a tool for designing devices of performances superior to the best reported to date, thus closing in on fundamental limits. The model is employed to design a graded-interface 8.1 µm-emitting QCL with suppressed carrier leakage via conduction-band engineering, which reaches a maximum front-facet wall-plug efficiency value of 22.2 %, significantly higher than the current record (17 %); thus, a value close to the fundamental front-facet, upper limit (i.e., 25 %) for ∼8 µm-emitting QCLs.

## 1 Introduction

Conventionally, studies of mid-infrared (IR) QCLs have been performed considering abrupt interfaces characterized by two IFR-scattering parameters: the root-mean square (RMS) height Δ, and the in-plane correlation length Λ [1]. The two IFR parameters had to be obtained from empirical fits to experimental data [2], [3], [4] since, although well/barrier interfaces were known to be graded, there was no experimental method for measuring the actual interfaces’ characteristics. Furthermore, the electro-optical characteristics of mid-IR QCLs of record-high performance [5], [6], as far as maximum output power and wall-plug efficiency (WPE), could not be theoretically reproduced by using abrupt-interfaces modeling. Thus, the reasons behind those devices’ excellent performances are poorly understood, preventing both finding out how they worked as well as how to design QCLs of even higher performance.

A breakthrough occurred 3 years ago with the publication of a generalized, nonequilibrium Green’s functions (NEGF)-based IFR-scattering formalism for modeling terahertz (THz) QCLs with graded interfaces [7]. Besides considering Δ and Λ, two other IFR parameters were introduced: the graded-interface width *L* and an axial correlation length Δ_⊥_. Furthermore, it was shown, for graded-interfaces Ge/Ge–Si QCL structures designed for THz operation, that the four IFR parameters can be obtained from analysis of results of atom-probe tomography (APT). Here, we generalize this theory to the multiband case, which is needed to accurately describe mid-IR QCLs. This latest theory has already been used [8] to obtain, via APT analysis, the IFR parameters of one of our step-tapered active-region (STA)-type [9] 4.6 µm-emitting InGaAs/AlInAs QCL structures grown by metal–organic chemical vapor deposition (MOCVD). The APT analysis revealed: (a) Δ values of ∼0.135 nm at the interfaces of moderately strained barriers, which have almost identical strain as the moderately strained barriers in the published 4.9 µm-emitting QCL; (b) that the Δ value increases with the barriers’ Al content; (c) Λ values of ∼6 nm; i.e., shorter than ∼9 nm value inferred from abrupt-interface studies [2], [3]; and (d) *L* values of ∼0.55 nm. Subsequently, we used our model with graded interfaces to design STA-type 4.6 µm-emitting QCLs with those APT-obtained IFR parameters. QCL structures were grown by MOCVD, and results from processed devices were found to be in very good agreement with those predicted by the model (see Supplementary Material, section A). That validated not only our model with graded interfaces but also the IFR-parameter values obtained via APT.

Here we show that, guided by our findings from APT analysis of 4.6 µm-emitting InGaAs/AlInAs QCLs, when we apply the NEGF-based IFR-scattering theory for graded interfaces (with Λ = 6 nm and Δ = 0.13 nm) to 4.9 µm-emitting [5] as well as (with Λ = 6 nm) to 8.3 µm-emitting [6] QCL structures of record front-facet peak-pulsed WPE values: 27 % and 17 %, respectively, we can deduce the remaining IFR parameters’ values by reproducing the experimental threshold-current density, *J*_*th*_, values and voltage–current (V–I) curves. The light–current (L–I) curves are found to be accurately reproduced as well.

As a result, the key device-design features needed for achieving high-performance mid-IR QCLs, at room temperature, are, for the first time, revealed. In particular, photon-induced carrier transport [10], [11], [12], [13] (PICT) needs to be implemented for achieving both high-power and high-WPE operation. However, it is also found that these record-performance devices have a significant amount (23–28 %) of normalized carrier leakage, *J*_leak_/*J*, where *J*_leak_ is the leakage-current density and *J* is the current density at threshold [14] and/or close above threshold [15].

Then, the model considering graded interfaces can be used as a tool for designing higher performance QCLs by suppressing carrier leakage via the STA-QCL approach [9]. As an example, an 8.1 μm-emitting STA-type QCL is designed with basically the same graded-interface IFR parameters as those we found for 8.3 µm-emitting QCLs, and with PICT action. The obtained maximum WPE value is significantly higher than that for 8.3 μm-emitting QCLs (i.e., 22.2 % vs. 17 %), thus approaching the fundamental upper limit [16] of ∼25 % for ∼8 μm-emitting QCLs.

## 2 High front-facet wall-plug efficiency 4.9 μm-emitting QCL

The device studied is of the so-called shallow-well design [5], which was shown [9] to be of the (linear)-tapered active-region (TA) type, in that its active-region (AR) barrier heights increase linearly from the conventional-injection barrier to the exit barrier. The front-facet maximum pulsed WPE value is 27 %, at 298 K heatsink temperature; that is, about twice the room-temperature, front-facet maximum values for 4.6–5.0 μm-emitting, nonresonant-extraction [17] (i.e., 15.1 %) and STA [4] (i.e., 14 %) QCL types. As such, it represents the highest room-temperature pulsed WPE value reported to date for any type of QCL capable of CW operation [16], [18].

### 2.1 NEGF-based analysis

As mentioned above, the NEGF formalism for modeling with graded interfaces, which was initially developed for THz-QCL structure analysis [7], was modified for use in mid-IR QCL analysis, as discussed below. Specifically, the model was extended to *k*⋅*p* multiband theory and modified to take into account the variation of the effective mass in the graded regions, as well as the Δ values for each graded interface. The inputs to the model for a given QCL structure are as follows: the layer compositions and nominal thicknesses for one stage; the injector sheet-doping density; the sum of the waveguide loss, *α*_w_, and mirror loss, *α*_m_; the optical-mode confinement factor to the core region, Γ; and the values for the four IFR parameters. Using the experimentally measured [8] 6 nm value for Λ and 0.135 nm for Δ of moderately strained barriers, the other two IFR parameters were deduced by matching the *J*_th_ value and the V–I curve, while taking into consideration that Δ increases with the barriers’ Al content [8]. Then, we get: Δ = 0.10 nm at the lattice-matched barrier interfaces, 0.13 nm at the moderately strained barriers’ interfaces, and 0.17 nm at the heavily strained AlAs barriers’ interfaces; *L* = 0.4 nm; and Δ_⊥_ = 0.1 nm. The different Δ values are consistent with the trend, found from APT-data analysis [8], that Δ increases with the layer’s relative strain with respect to the InP substrate, and the fact that AlAs layers may have residual oxygen incorporation. In particular, the Δ value at the moderately strained barriers’ interfaces (i.e., 0.13 nm) is similar to that found via APT (i.e., ∼0.135 nm) for almost identical barriers (i.e., Al_0.64_In_0.36_As vs. Al_0.65_In_0.35_As), while the value at the AlAs barriers’ interfaces (i.e., 0.17 nm) is smaller than that found via APT (i.e., 0.2 nm), most likely due to the crystal growth method used [i.e., gas-source molecular beam epitaxy (GS-MBE)], which should have less residual oxygen incorporation than MOCVD, given to ultrahigh-vacuum chambers employed in MBE-growth reactors.

**Figure 1:**
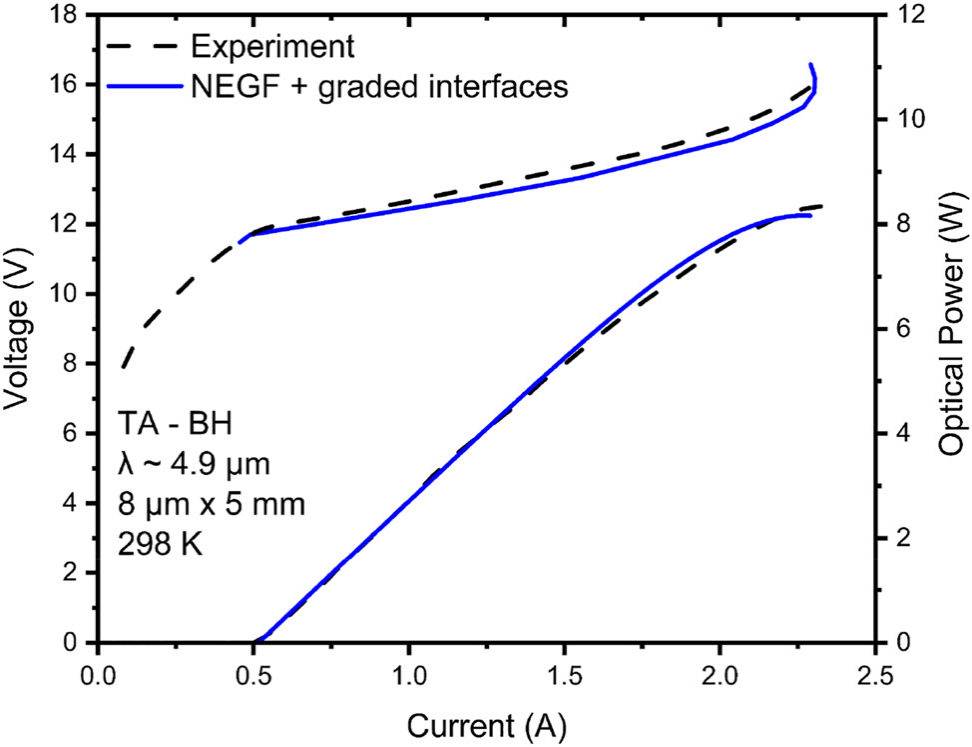
Voltage– and light–current curves (500 ns pulse width; 5 % duty cycle) of 4.9 μm-emitting TA-type QCL [5], [9] versus those obtained by using NEGF modeling with graded interfaces. The employed graded-interface IFR-scattering parameters are: Δ = 0.10 nm, 0.13 nm, and 0.17 nm for the interfaces at the lattice-matched barrier, moderately strained barriers, and AlAs barriers, respectively; Λ = 6 nm; *L* = 0.4 nm; and Δ_⊥_ = 0.1 nm.

The experimental V–I and L–I curves are compared to those generated via the NEGF model with graded interfaces in Figure 1. The differential resistance *R*_diff_ has a 1.6 Ω calculated value, quite close to the 1.7 Ω experimental value, and the maximum current density, *J*_max_, is basically the same in both cases: 5.76 kA/cm^2^ versus ∼5.75 kA/cm^2^. The L–I curve is well approximated to the maximum peak pulsed front-facet emitted power of ∼8 W. To the best of our knowledge, this is the first time that the characteristics of the record wall-plug efficiency QCL [5] have been theoretically reproduced.

We note that the *R*_diff_ value is ∼50 % of the value obtained [19] for conventional, 40-stage 4.8 μm-emitting QCLs (i.e., 3.5 Ω) of same buried-ridge dimensions (i.e., ∼8 μm × 5 mm). Furthermore, subtracting the calculated cladding resistance value of ∼0.5 Ω, the core-region *R*_diff_ value is ∼40 % that for the conventional QCL (i.e., 1.2 Ω vs. 3 Ω). The difference can only be explained by the fact that above threshold there is significant PICT action [10], [11], [20]; that is, virtually all carrier transport is photon induced. The onset of PICT action is evidenced by the sharp “kink” in the V–I curve at threshold. The other telltale sign of PICT action is that, for the same injector sheet-doping density, *n*_s_ (i.e., 0.9 × 10^11^ cm^2^), the *J*_max_ value is ∼1.5 times the value for conventional QCLs (i.e., 3.8 kA/cm^2^) [21]. This is typical of PICT-action QCLs, which have significantly shorter transit time *τ*_*trans*_ than conventional QCLs, since carrier transport in a PICT-action QCL is in large part limited by the photon-assisted tunneling time between the upper-laser (*ul*) and lower-laser (*ll*) levels [11], while for a conventional QCL *τ*_*trans*_ involves the electrically driven average transport time through one stage [20], [22]. For this device, since $J_{\max}=\frac{en_{s}}{\tau_{\mathrm{trans}}}$ [22], the *τ*_*trans*_ value at shutoff decreases from ∼3.8 ps for conventional 4.9 μm-emitting QCLs [21] to ∼2.5 ps. Therefore, for this PICT-action device, the combination of lower voltage values above threshold and increased dynamic range causes the maximum WPE value to become significantly higher than for conventional QCLs.

**Figure 2:**
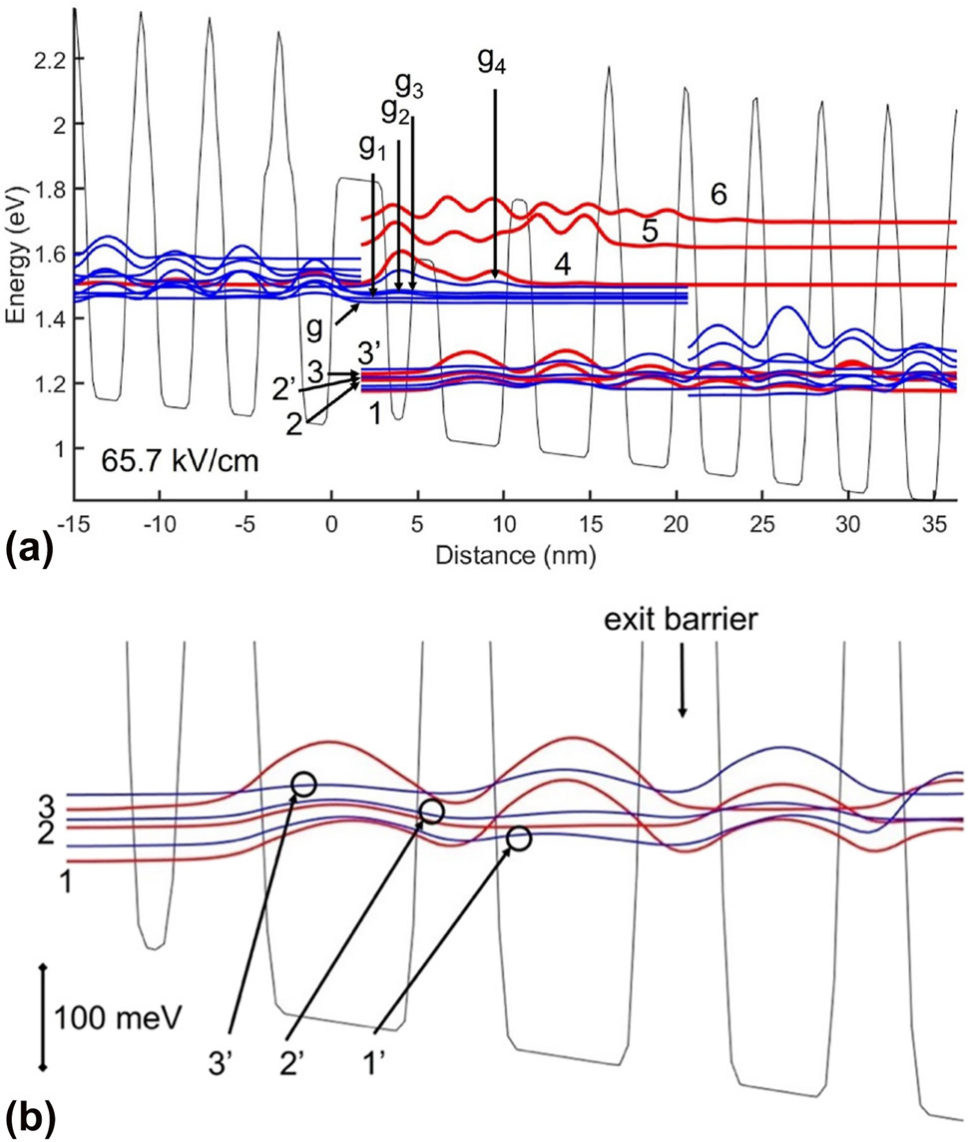
Conduction-band diagram and relevant wavefunctions for the 4.9 μm-emitting TA-type QCL [5], at threshold: (a) for the whole stage; (b) for the low-energy states in the active region (i.e., states 3, 2, and 1) and the extractor states penetrating the active region (i.e., states 3′, 2′, and 1′). States 3 and 3′ are the lower laser levels.

Strong PICT action, as originally described by Blaser et al. [10] and Choi et al. [11], involved a QCL for which the injector ground state was also the *ul* level, thus a strong diagonal transition was involved. However, for state-of-the-art QCLs, PICT action requires strong coupling (7–10 meV) between the injecting state and the *ul* level, a strong diagonal transition, and a very short *ll*-level lifetime, which, in turn, ensures quick gain recovery. Figure 2 shows, at threshold, the schematic conduction-band (CB) diagram with graded interfaces and the relevant wavefunctions: (a) for the whole stage and (b) for the low-energy states in the AR. We observe that: (a) states 2 and 2′ in the prior-stage AR correspond to states *g*_4_ and 4, and (b) states 2 and 2′ have the same strong coupling (7.5 meV), at a resonance field of 66.8 keV (i.e., a detuning from threshold of only 1.1 kV/cm), as states *g*_4_ and 4 do. Furthermore, as discussed below, at resonance the lasing transition primarily occurs from level *g*_4_, while above resonance level 4 becomes the *ul* level. Therefore, we conclude that, unlike in conventional QCLs, in QCLs with strong PICT action injection into the *ul* level occurs at the exit barrier of the prior stage via tunneling, in the presence of strong scattering, from a low-energy AR state; that is, similar to what happened in the 3-level system analyzed by Choi et al. [11], except that the low-energy AR state is not the *ll* level. This direct injection from level 2 of the prior stage ensures a low applied field value at threshold, *F*_th_, (i.e., 65.7 kV/cm) unlike conventional QCLs for which, after injection through the prior-stage exit barrier, electrons are scattered down to the injector ground state, *g*, and lasing starts at a higher field when *g* and the *ul* level reach the detuning value necessary for achieving population inversion via resonant tunneling injection [22]. That is, for PICT-action devices there is no resonant tunneling injection needed to achieve population inversion [11].

**Figure 3:**
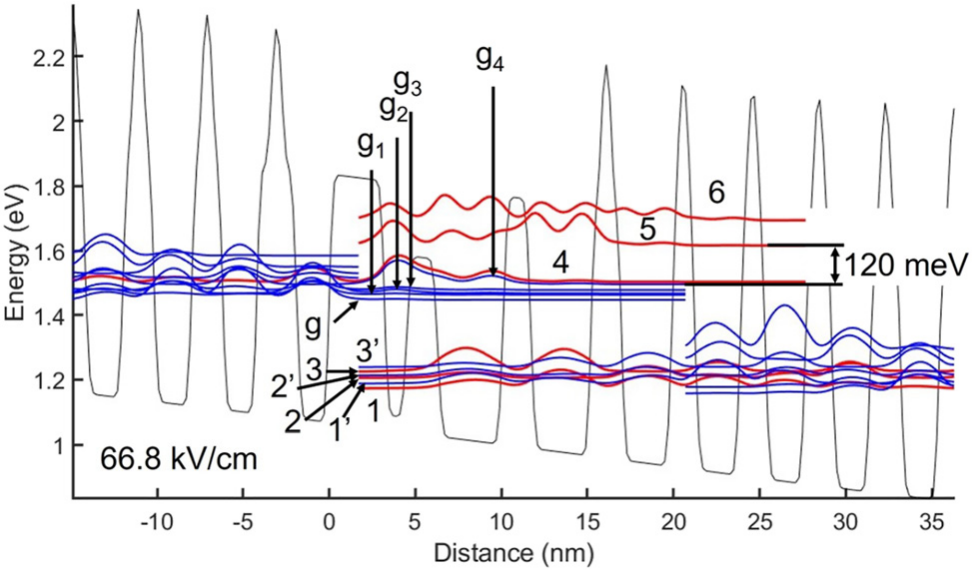
Conduction-band diagram and relevant wavefunctions for the 4.9 μm-emitting TA-type QCL [5], at 1.4 times threshold. *g*_4_ is the upper laser level. Prior-stage states 2 and 2′, and states *g*_4_ and 4 simultaneously reach their respective resonance.

From Figures 2(a) and 3 (i.e., at resonance), it is clear that there is a strong diagonal transition, and that at resonance states *g*_4_ and 4 become degenerate, in that their wavefunctions strongly overlap.

As pointed out above, lasing starts via injection from state 2 of the prior stage when that state and extractor state 2′ are very close to their resonance. (We will see later for the designed 8.1 μm-emitting QCL, which has stronger PICT action, that lasing starts right at the resonance of states 2 and 2′). Such injection allows lasing onset at a relatively low applied field, *F*_th_, which, in turn, allows for both a low threshold-voltage value as well as low thermal backfilling, as needed for efficient CW operation [23]. The situation looks similar to excited-state injection, originally called pocket injection [24], from a relatively high-energy injector-miniband state, but, given that state *g*_4_ is the *ul* level at resonance, this is not an excited-state injection scheme.

The second condition for PICT action: strong diagonal transition [10], [11], [13], is also met. In this case, just as for STA-type QCL [4], [25], there is resonant extraction from the *ll* level, state 3. That is, state 3 strongly couples to the extractor state 3′, with an energy splitting of 10.5 meV at 72.6 kV/cm. This means that up to 72.6 kV/cm extractor state 3′ is another *ll* level. Thus, at threshold and at the *g*_4_/4 resonance, there are lasing transitions to both *ll* levels 3 and 3′. However, for this particular device, we find that a dominant gain peak emerges only at resonance (i.e., at 1.1 kV/cm above *F*_th_), which corresponds to a drive level of 1.4 × *J*_*th*_, with the *ul* level being state *g*_4_. The respective dipole matrix elements are *z*_*g*4,3_ = 5.9 Å and *z*_*g*4,3^′^_ = 3.2 Å, which give an overall matrix element *z*_*g*4,ll_ = 6.7 Å; that is, a strong diagonal transition.

The third condition for PICT action: quick depopulation of the *ll* level [11], occurs for state 3: (a) via tunneling into extractor state 3′; (b) via relaxation to states 2, 2′, 1, and 1′ followed by tunneling into extractor states 2′ and 1′ (see schematic CB diagram and relevant wavefunctions in Figure 2(b)). The global *ll*-level lifetime, *τ*_*ll*,*g*_, characterizing relaxation to states 2, 2′, 1, and 1′, has a low value of 0.07 ps [26], typical of QCL structures with tall barriers on the downstream side of the AR [4], [14], in which case IFR scattering dominates (e.g., for this device the IFR component of *τ*_*ll*,*g*_ is 0.10 ps). Extraction from levels 3, 2, and 1 is ensured by strong coupling to states 3′, 2′, and 1′: 10.5 meV, 7.5 meV, and 10.6 meV, respectively. Then, the low-energy AR states (i.e., 1–3) together with the penetrating extractor states (i.e., 1′-3′) form a relatively wide (∼70 meV) miniband; thus, ensuring highly efficient extraction, just like in bound-to-continuum QCLs [27], as needed for quick gain recovery; that is, quick replenishing of the *ul* level population to compensate for its depletion via stimulated emission.

**Figure 4:**
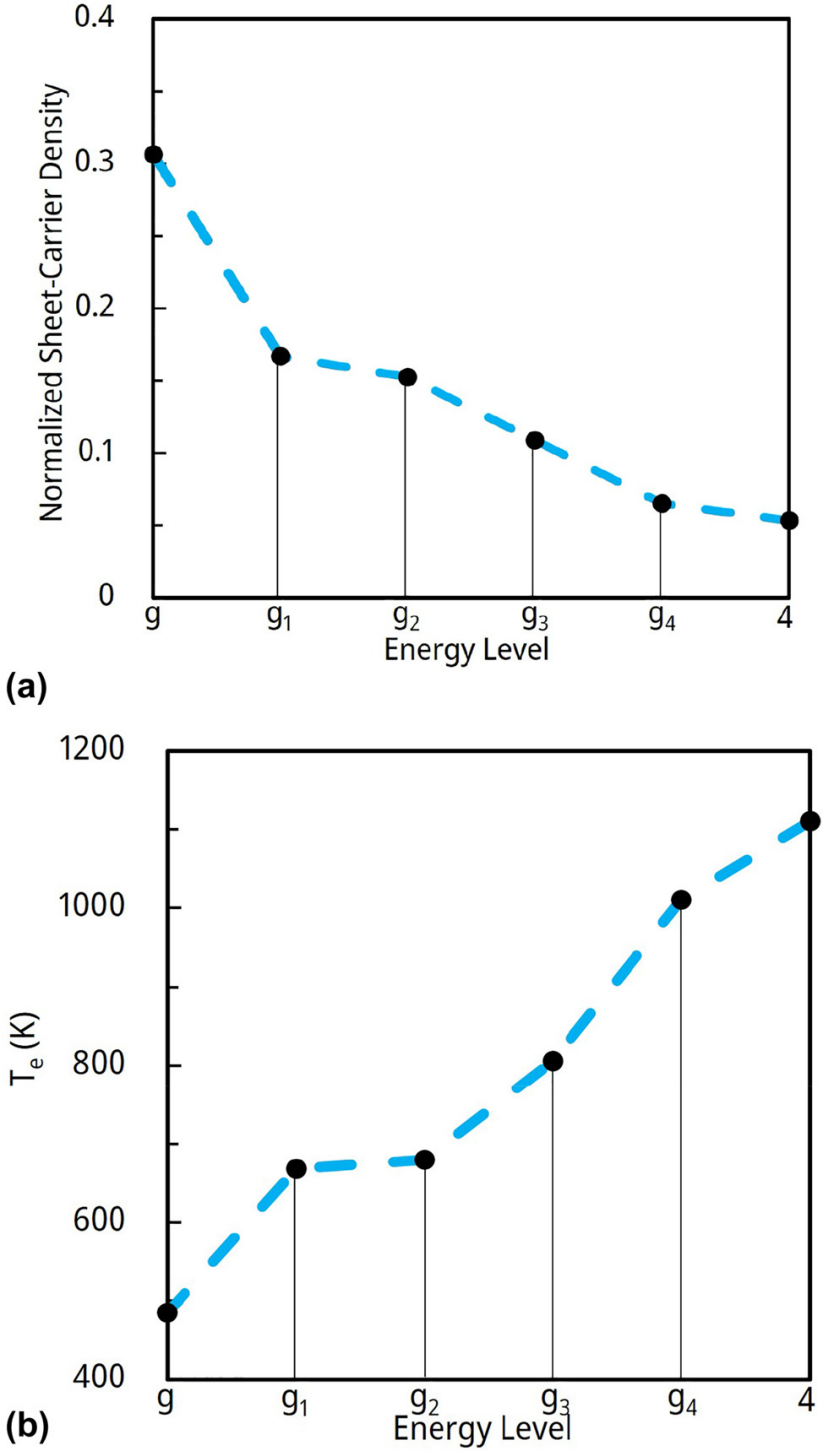
NEGF-analysis results for the 4.9 um-emitting QCL at the *g*_4_/4 resonance: (a) normalized sheet-carrier densities and (b) electron temperatures, in relevant energy levels.

NEGF analysis also provides the sheet-carrier densities as well as the electron temperatures in each subband of interest [3]. We show in Figure 4: (a) the normalized sheet-carrier densities and (b) the electron temperature (at 1.4 × *J*_*th*_), *T*_e_, values for energy states *g*, *g*_1_, *g*_2_, *g*_3_, *g*_4_, and 4. Notably the sheet-carrier densities in states *g*_4_ and 4 are almost identical, as expected given that we are at the resonance between the two states. The electron temperatures generally increase with state energy, that is, a nonthermal population exists, as previously observed from the NEGF analysis of 8.5 μm-emitting QCLs [3]. In particular, the *T*_e_ values for states *g*_4_ and 4 are quite high (1060 K on the average); that is, much higher than previously taken for thermalized subbands in the injector miniband [14]. As we shall see, these high *T*_e_ values play a critical role in determining the carrier-leakage currents triggered by LO-phonon and IFR scattering from the *ul* level and the other states.

Finally, the global *ul*-level lifetime, *τ*_*ul*,*g*_, has a high value of 2.3 ps, due to both the strong diagonal transition and the short (i.e., lattice-matched) barrier. The high *τ*_*ul*,*g*_ value together with the low *τ*_*ll*,*g*_ value lead to a high lasing-transition efficiency [4] *η*_*tr*_ value of 97 %, which is another reason behind the record WPE value. However, since we are at 1.4 × *J*_*th*_, the *τ*_*ul*,*g*_ value is affected by the stimulated-emission lifetime, *τ*_*stim*_, which we calculate to be 3.16 ps (see Supplementary Material, section C). Then, *τ*_*ul*,*g*_ decreases to 1.33 ps, and the lasing-transition lifetime *τ*_*ul*,*ll*_ decreases from 5.29 ps to 1.98 ps, and, assuming that *τ*_*ll*,*g*_ (0.07 ps) hardly changes, the *η*_*tr*_ value decreases to 94.8 %.

### 2.2 Elastic scattering and carrier-leakage analysis

NEGF analysis provides the *F*_th_ and resonance-field values (Figures 2 and 3), the sheet-carrier densities and the electron temperatures in relevant states and in the *ul* level (e.g., Figure 4 for the 4.9 μm-emitting QCL at resonance). Then, one can apply the previously developed comprehensive carrier-leakage formalism [14]; although, as discussed below, it is valid only at threshold, thus it is strictly accurate only for the 8.3 μm- and 8.1 μm-emitting QCLs described in Sections 3 and 4. We employ a 3-band *k*⋅*p* solver to match the bands extracted from NEGF analysis. However, for calculating relevant lifetimes, one needs to consider the graded nature of the interfaces. Extending the theory of scattering by rough and graded interfaces [7] to the multiband case, we generalize the expression for the IFR scattering rate between a state *m* and a lower-energy state *n*, in heterostructures of structures of varying barrier and well compositions:

(1a) $$\begin{array}{ll} \frac{1}{\tau_{mn}^{\mathrm{IFR}}} & =\frac{2\pi }{\hbar }\underset{\vec{q}}{\sum }\frac{\pi \Lambda^{2}}{S}e^{-\frac{\Lambda^{2}q^{2}}{4}}|V_{mn}^{\left(z\right)}{|}^{2}\\ & \;\times \delta \left(E_{m}\left(\vec{k}\right)-E_{n}\left(\vec{k}+\vec{q}\right)\right) \end{array}$$

(1b) $$|V_{mn}^{\left(z\right)}{|}^{2}=\Delta^{2}\left(z\right)\underset{b}{\sum }\left(\begin{array}{c} \int dz_{1}\int dz_{2}\varphi_{m,b}^{*}\left(z_{1}\right)\varphi_{m,b}\left(z_{1}\right)\varphi_{n,b}^{*}\left(z_{2}\right)\varphi_{n,b}\left(z_{2}\right)\\ \frac{\partial \bar{V_{b}}}{\partial z}\left(z_{1}\right)\frac{\partial \bar{V_{b}}}{\partial z}\left(z_{2}\right)e^{-\left|z_{2}-z_{1}\right|\;/\Lambda_{⊥}} \end{array}\right)$$

where $\bar{V_{b}}$ is the in-plane-averaged potential associated with the band *m*, $\varphi_{m,b}\left(z\right)$ is the component in band *b* of the *m*-state wavefunction along the growth direction, $\Delta \left(z\right)$ is the RMS height of isoconcentration surfaces, $\vec{k}$ and $\vec{q}$ are the initial and exchange in-plane wavevectors, respectively, *z* denotes the growth axis, and *S* is a normalization surface. The corresponding self-energies are implemented into the NEGF code, providing a full simulation of the simultaneous effects of roughness and grading. However, for the sake of the analysis, when the different interfaces are well separated with respect to the interfacial width, a scattering-rate reduction *F* factor due to graded interfaces can be defined [7]:

(2a) $$|V_{mn}^{\left(z\right)}{|}^{2}=F\underset{i,b}{\sum }\Delta_{\text{i}}^{2}\int dz_{1}\int dz_{2}\varphi_{m,b}^{2}\left(z_{i}\right)\varphi_{n,b}^{2}\left(z_{i}\right){\left|\delta V_{bi}\right|}^{2}$$

(2b) $$F=\exp\left[\frac{L^{2}}{16\ln\left(2\right)\Delta_{⊥}^{2}}\right]\left[erf\left[-L/\left(4\sqrt{\ln\left(2\right)}\Delta_{⊥}\right)\right]+1\right]$$

where Δ_*i*_ is the RMS height at the *i*th interface and *δV*_*bi*_ is the CB offset at the *i*th interface.

For transitions from the *ul* level, *m* is the *ul*-level state number and *n* is the *ll*-level state number or the state number of any of the rest of low-energy AR and extractor states. For transitions from the *ll* level(s), *m* is the *ll*-level state number and *n* is the state number of any of the lower-energy AR and extractor states. For example, for the band diagram and wavefunctions shown in Figure 2, *m* = *g*_4_ and *n* = 3, 3′, 2, 2′, 1, and 1′ for transitions from the *ul* level, state *g*_4_; while *m* = 3 [16], and *n* = 2, 2′, 1, and 1′ for transitions from the *ll* level; where the primed states are extractor states. In the general 3-band case, the effective-mass description is not appropriate anymore, unless defining energy-dependent effective masses. However, since we found that using energy-dependent effective masses negligibly affects the IFR scattering rates compared to when considering effective masses adjusted for strain in each quantum well, we are using an approximate expression:

(3) $$\begin{array}{ll} \frac{1}{\tau_{mn}^{\mathrm{IFR}}} & ≅\frac{\pi }{\hbar^{3}}\Lambda^{2}F\underset{i}{\sum }m_{ci}\Delta_{i}^{2}\delta V_{i}^{2}\varphi_{m}^{2}\left(z_{i}\right)\varphi_{n}^{2}\left(z_{i}\right)\exp\left(-\frac{\Lambda^{2}m_{ci}E_{mn}}{2\hbar^{2}}\right) \end{array}$$

where *m*_ci_ is the effective mass at the *i*th interface, *δV*_*i*_ is the CB offset at the *i*th interface, and *φ*_*ul*_ (z_i_) and *φ*_*ll*_(z_i_) are the wavefunction amplitudes of the *ul* or *ll* levels at the *i*th interface. *E*_mn_ represents the energy difference between states, and Λ is taken to be the same at all interfaces, as observed from APT analysis [8].

For the QCL structure shown in Figure 2 (i.e., for *L* = 0.4 nm and Δ_⊥_ = 0.1 nm) *F* has a value of 0.38. As pointed out in [7], the reduction of the scattering rate for graded interfaces versus abrupt interfaces can be understood in view of the fact that a graded interface does not behave like a single scattering center, but instead acts as a collection of different, only partially correlated, scattering centers. That is, the degree of IFR scattering coherence is significantly reduced for graded interfaces versus abrupt interfaces.

The AD scattering rate between two selected states *m* and *n*, in a given ungraded well or barrier layer, is given in [14]. However, for the graded regions between ternary-alloy well and barrier layers, the AD scattering rate expression is quite complicated since it has to consider quaternary alloys composed of three group III elements and one group *V* element. Then, for a given Al_*x*_In_*y*_Ga_1−*x*−*y*_As graded interface, the total scattering rate between two selected states *m* and *n* is given by:

(4a) $$\begin{array}{ll} \frac{1}{\tau_{mn}^{AD}} & =\frac{1}{8}\int_{0}^{L}\frac{m_{c}\left(z\right)a{\left(z\right)}^{3}}{\hbar^{3}}\left[x\left(z\right)\left[1-x\left(z\right)-y\left(z\right)\right]\left[1-y\left(z\right)\right]\right.\\ & \;\times {\left(V_{Al-Ga}\right)}^{2}+y\left[1-x\left(z\right)-y\left(z\right)\right]\left[1-x\left(z\right)\right]\\ & \;\times {\left(V_{In-Ga}\right)}^{2}+x\left(z\right)y\left(z\right)\left[x\left(z\right)+y\left(z\right)\right]{\left(V_{Al-In}\right)}^{2}\\ & \;+2x\left(z\right)y\left(z\right)\left[1-x\left(z\right)-y\left(z\right)\right]\left(V_{Al-Ga}V_{In-Ga}\right.\\ & \;\left.\left.+V_{Al-Ga}V_{Al-In}-V_{In-Ga}V_{Al-In}\right)\right]\varphi_{m}^{2}\left(z\right)\varphi_{n}^{2}\left(z\right)\\ & \;\times dz \end{array}$$

where *V*_*Al*−*Ga*_ = 0.8 eV, *V*_*Al*–*In*_ = −0.6 eV, and *V*_*In*−*Ga*_ = 1.4 eV are the differences between the CB minima of the binary-alloy components, and *a*, the lattice parameter, is graded in accordance with Vegard’s law:

(4b) $$a\left(z\right)=y\left(z\right)a_{\mathrm{InAs}}+\left[1-x\left(z\right)-y\left(z\right)\right]a_{\mathrm{GaAs}}+x\left(z\right)a_{\mathrm{AlAs}}$$

The integral is nontrivial to solve, so we approximate the graded interface in 0.1 nm-wide rectangular steps of compositions equal to those at the end of each step. Then, for each 0.1 nm-wide step, we calculate:

(5) $$\begin{array}{ll} {\left.\frac{1}{\tau_{mn}^{AD}}\right|}_{s} & =\frac{1}{8}\frac{m_{cs}a_{s}^{3}}{\hbar^{3}}\left[x_{s}\left(1-x_{s}-y_{s}\right)\left(1-y_{s}\right){\left(V_{Al-Ga}\right)}^{2}\right.\\ & \;+y_{s}\left(1-x_{s}-y_{s}\right)\left(1-x_{s}\right){\left(V_{In-Ga}\right)}^{2}\\ & \;+x_{s}y_{s}\left(x_{s}+y_{s}\right){\left(V_{Al-In}\right)}^{2}+2x_{s}y_{s}\\ & \;\times \left(1-x_{s}-y_{s}\right)\left(V_{Al-Ga}V_{In-Ga}+V_{Al-Ga}\right.\\ & \;\times \left.\left.V_{Al-In}-V_{In-Ga}V_{Al-In}\right)\right]\varphi_{ms}^{2}\varphi_{ns}^{2}\times 0.1\;\text{nm} \end{array}$$

where *s* is the step number (e.g., if *L* = 0.4 nm the calculations are done for *s* = 1, 2, 3, and 4), *x*_*s*_, *y*_*s*_ are the quaternary-alloy Al and In fractions at the interface between step *s* and step *s* + 1, and $\varphi_{ms}^{2},\varphi_{ns}^{2}$ are the probability values of states *m* and *n* at the interface between step *s* and step *s* + 1. The profiles for the graded interfaces, as displayed for instance in Figure 2, follow an error function defined in [7], which is used to determine the lattice constant, *a*_s_, and the alloy fractions, *x*_*s*_, and *y*_*s*_, at each step.

**Figure 5:**
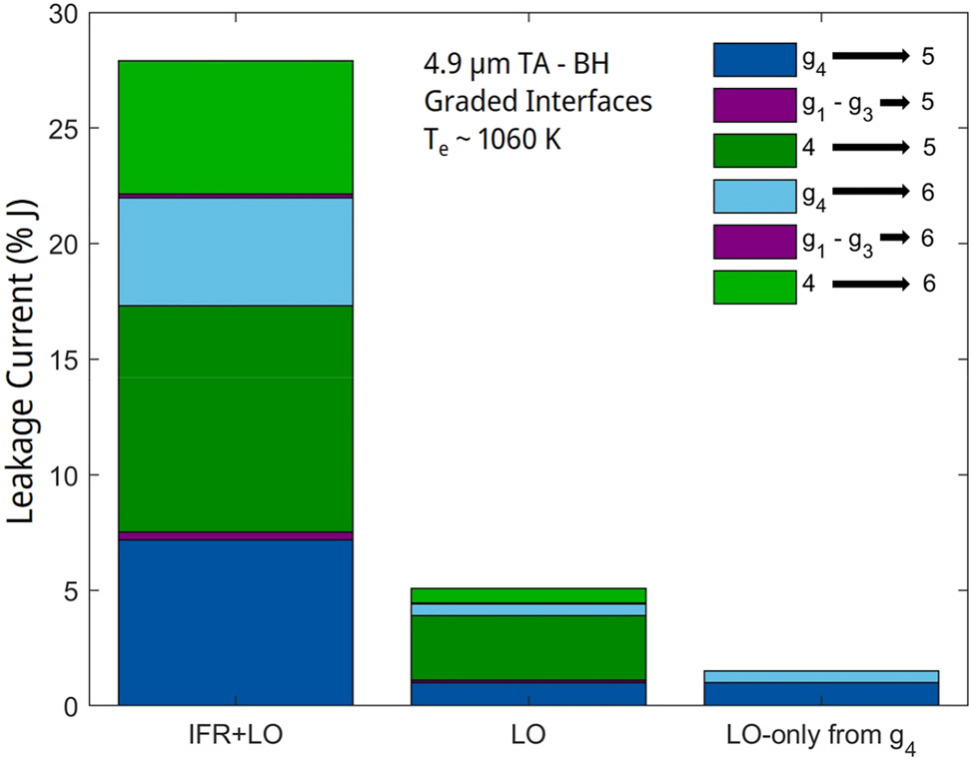
Bar graphs of the components of the relative leakage-current density through the active-region energy states 5 and 6, for the 4.9 μm-emitting TA-type QCL [5]. LO, and LO-only from *g*_4_ stand for leakage triggered only by LO-phonon scattering in the presence of elastic scattering, and leakage only from the *ul*-level, state *g*_4_, in the absence of elastic scattering, respectively.

The LO-phonon- and IFR-triggered leakage-current densities, $J_{\mathrm{leak}}^{LO}$ and $J_{\mathrm{leak}}^{\mathrm{IFR}}$ , from both the *ul* level and relevant states through high-energy AR states down to low-energy AR states [23] are calculated as in [14], except that, given the relatively high electron temperatures found in this study, the exponential term in the $J_{\mathrm{leak,mn}}^{LO}$ expression [i.e., $\exp\left(-\frac{E_{nm}}{kT_{em}}\right)$ ] has to be replaced with [28]: $\exp\left\{-\frac{\left(E_{nm}+\hbar \omega_{LO}\left[\left(\frac{T_{em}}{T}\right)-1\right]\right)}{kT_{em}}\right\}$ , where *T*_em_ is the electron temperature in state *m*, *T* is the lattice temperature, and *ℏω*_*LO*_ is the phonon energy. We show in Figure 5 a bar chart of the following: the total *J*_*leak*_ value normalized to *J*; the total normalized $J_{\mathrm{leak}}^{LO}$ value; and (c) the total normalized $J_{\mathrm{leak}}^{LO}$ value in case of inelastic scattering only from the *ul* level, state *g*_4_. The calculation is done at resonance (i.e., at a field of 66.8 kV/cm), where *J* = 1.4 × *J*_*th*_, since there the gain has one dominant peak, while $\frac{J_{\mathrm{leak}}}{J}≅\frac{J_{\mathrm{leak,th}}}{J_{th}}$ for *J* values close above threshold [9], [23].

Leakage through the high-energy states 5 and 6 is mostly IFR triggered (i.e., the IFR part is 82 % of the total leakage) and occurs mostly from the state *g*_4_ and state 4. This is understandable, given the high average electron temperatures (1060 K) in those energy states. The inelastic-only leakage from the *ul* level, considered in early work [28], is only 5.4 % of the total normalized leakage (i.e., 1.5 % vs. 28 %) proving, just as in the case of abrupt-interface calculations [14], that elastic scattering dominates carrier leakage in mid-IR QCLs. In this case, level 4 acts like a parasitic AR state, just 7.5 meV above the *ul* level. There is more leakage from it than from level *g*_4_, since it is 100 K hotter (i.e., 1110 K vs. 1010 K). Notably, the total normalized leakage through state 5 is basically the same as that calculated via abrupt-interfaces analysis of the same QCL structure [14] (i.e., 17.5 % vs. 18.5 %), despite the inherent reduction in IFR scattering rates for graded-interfaces structures [7] (i.e., *F* = 0.38) and a higher energy difference between states 5 and 4, 5 and *g*_4_ (i.e., 113 and 120 meV), and states 5 and 4 for the latter case (i.e., 79 meV). We attribute this primarily to much higher electron temperatures (i.e., 1060 K average vs. 347 K) as a result of employing NEGF-based modeling.

However, as pointed out above, we have to take into account the stimulated lifetime, in which case we estimated an *η*_*tr*_ value of 94.8 %. If there is no leakage and taking unity tunneling injection efficiency, the *total* injection efficiency [4], *η*_inj_, becomes unity. Then, the fundamental limit for the internal efficiency, *η*_*i*_, is the *η*_*tr*_ value (94.8 %). Since the experimentally measured *η*_*i*_ value was 70 % [5], the differential pumping efficiency [9], [23], *η*_*p*_ = 1 − *J*_*leak*_/*J*, had to be 73.8 %; thus, giving a total normalized leakage of 26.2 %. That is, considering stimulated emission, the normalized leakage is approximately 26 % at 1.4 × *J_th_*.

The relatively large normalized leakage values we find (i.e., 26–28 %) show that even for the QCL of record WPE value carrier leakage is quite significant. The differential pumping efficiency, *η*_*p*_, is 72–74 %, and the total injection efficiency *η*_inj_ is only 71–73 %. Given that *η*_*tr*_ = 94.8–97 %, in the ideal case that *η*_inj_ is unity, the fundamental upper limit for *η*_*i*_ is the *η*_*tr*_ value, which leads, at *λ* = 4.9 μm, to a WPE fundamental upper limit of 39–40 % [4]. Therefore, there is considerable room for further increasing the front-facet WPE value at *λ* ∼ 4.9 μm (i.e., from 27 % to values close to 40 %). That can be achieved by designing graded-interface STA QCL structures with PICT action *and* virtually complete carrier-leakage suppression [14].

### 2.3 Sensitivity analysis to variations in IFR parameters values

While keeping the Λ value constant at 6 nm, a sensitivity analysis to variations in IFR parameters values on device performance reveals that the slope efficiency, *η*_*sl*_, is the most sensitive device characteristic to variations in the *L*/Δ_⊥_ value and in the Δ value. Maximum errors of +/−4 % in the *η*_*sl*_ value have been chosen as the criterion for a reasonably good fit to experimental data. The *η*_*sl*_ value increases with increasing *L*/Δ_⊥_ value because less IFR-triggered carrier leakage is associated with increased *L*/Δ_⊥_ value (see Supplementary Material, section B). We don’t expect *L* to be wider than 0.55 nm or narrower than 0.30 nm. Thus, for the *L*/Δ_⊥_ value that best fits the experimental data (i.e., 4), the Δ_⊥_ value may well be in the 0.08–0.14 nm range. The *η*_*sl*_ value decreases with increasing Δ value because of increased IFR-triggered carrier leakage [14]. Details of the analysis are presented in Supplementary Material, section B. The findings confirm the importance of minimizing the IFR-triggered carrier leakage in order to maximize the device pulsed and CW performance [16].

### 2.4 Comparisons to results obtained with extracted abrupt-interface IFR parameters

Using Λ = 9 nm and Δ = 0.12 nm [2], the *J*_th_ value increases from 1.3 kA/cm^2^ to 2.2 kA/cm^2^, since injection occurs from the 1st-excited injector state into the *ul* level. There is no PICT action, as the device acts as a conventional QCL emitting at 4.5 μm (see Supplementary Material, section D).

## 3 High front-facet wall-plug efficiency 8.3 μm-emitting QCL

The device studied [6] has a conventional-like QCL structure (i.e., quantum wells and barriers of fixed compositions) and was designed for high WPE operation by: (a) employing a diagonal transition, to maximize the *ul*-level lifetime; (b) using a low value of 90 meV for the voltage defect at resonance, Δ_*inj,res*_ [16], [29]; and (c) having a low waveguide loss (1.34 cm^−1^). The front-facet maximum pulsed WPE value is 17 %, at 293 K heatsink temperature; that is, 1.6 times higher than the second-highest front-facet value reported to date (i.e., 10.6 % for an 8 μm-emitting STA QCL with injection from the 1st-excited injector state [30]) for QCLs emitting in the long-wave infrared (i.e., 8–12 μm wavelength).

### 3.1 NEGF-based analysis

**Figure 6:**
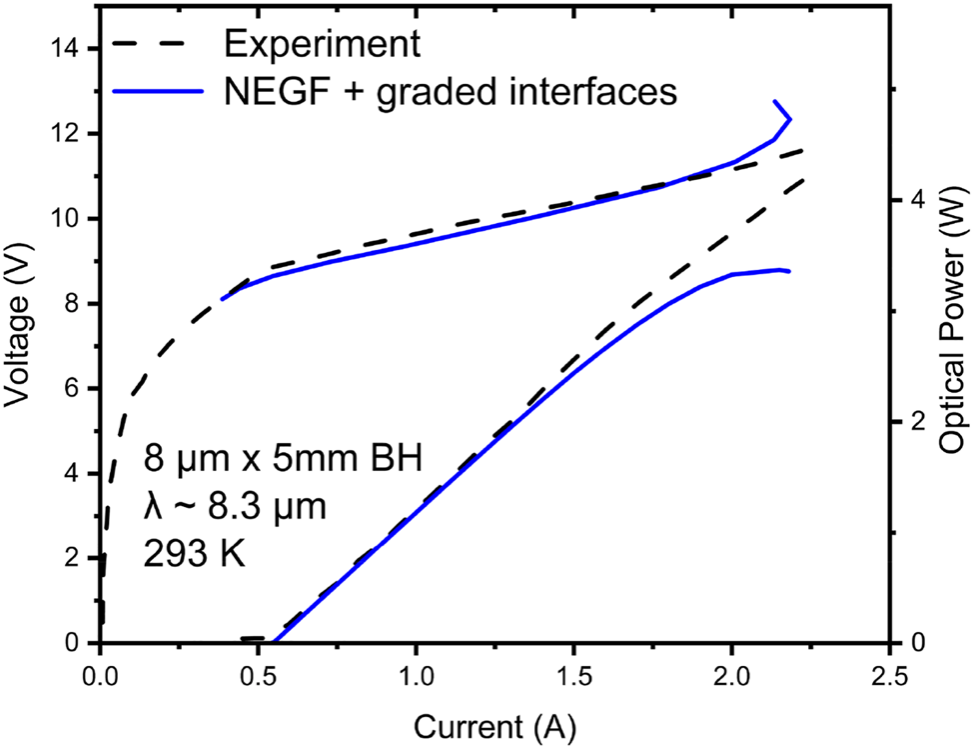
Voltage– and light–current curves of 8.3 μm-emitting QCL [6] versus those obtained by using the model with graded interfaces. The employed graded-interface IFR-scattering parameters are: Δ = 0.11 nm; Λ = 6 nm; *L* = 0.4 nm; and Δ_⊥_ = 0.1 nm.

We again consider the experimentally measured ∼6 nm value [8] for Λ. Then, just as for the 4.9 μm-emitting QCL, by matching the *J*_th_ value and the V–I curve, while considering values and trends found from APT analysis of mid-IR QCL structures, we obtain Δ = 0.11 nm, *L* = 0.4 nm, and Δ_⊥_ = 0.1 nm. The smaller Δ value than for the moderately strained barriers’ interfaces (i.e., Al_0.64_In_0.36_As/In_0.69_Ga_0.31_As) of 4.9 μm-emitting QCLs (i.e., 0.13 nm) may well reflect a lower degree of differential strain [8] for the employed barriers (i.e., Al_0.64_In_0.36_As/In_0.59_Ga_0.41_As). The experimental V–I and L–I curves are compared in Figure 6 to those generated via the NEGF-based model with graded interfaces. The calculated *R*_diff_ value: 1.65 Ω, is very close to the 1.6 Ω experimental value. However, the calculated *J*_max_ value (i.e., 5.45 kA/cm^2^) is lower than the experimental value (i.e., ∼5.7 kA/cm^2^). We suspect that this difference in *J*_max_ values reflects inadvertent overdoping of the grown QCL structure. The L–I curve matches the experimental curve very well to ∼3 × threshold. Despite the relatively small discrepancy in *J*_max_ values, to the best of our knowledge, this is the only theoretical reproduction to date of the electro-optical characteristics of the record wall-plug efficiency device [6] for long-wave infrared-emitting QCLs.

PICT action is evident from the fact the *R*_diff_ value is ∼60 % of the *R*_diff_ for a conventional 8.4 μm-emitting QCL [31] when considering the same pumped area (i.e., ∼8 μm × 5 mm). For the device’s published *n*_s_ value of 1.07 × 10^11^ cm^−2^, the *J*_max_ value for conventional 8.4 μm-emitting QCLs is ∼4.7 kA/cm^2^, given *τ*_*trans*_ ∼3.6 ps [21]; that is, the calculated *J*_max_ value is only ∼16 % higher than for conventional devices. This relatively low enhancement in *J*_max_ value is most likely due both to a moderate splitting at resonance between levels 2 and 2′ from the prior stage (i.e., 6.6 meV) as well as to a moderate degree of lasing-transition diagonality (i.e., *z*_ul,ll_ = 16.4 Å).

**Figure 7:**
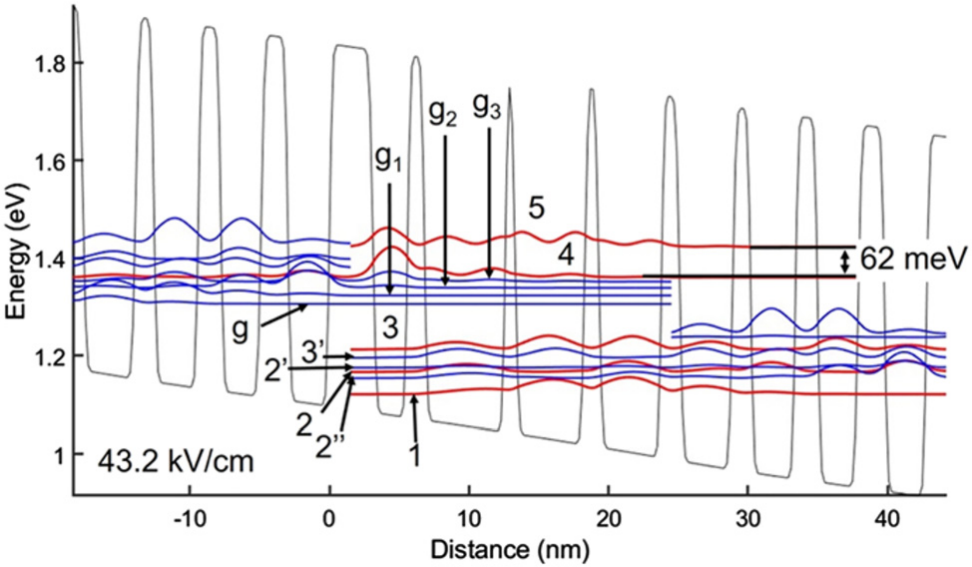
Conduction-band diagram and relevant wavefunctions for the 8.3 μm-emitting QCL [6], at threshold. Energy state 4 is the upper laser level.

A schematic representation of the CB diagram and relevant wavefunctions is shown in Figure 7. Injection occurs from the low-energy level 2, from the prior stage, directly into the *ul* level, state 4. As mentioned above, the states are moderately strong coupled (6.6 meV), and, just as for the 4.9 μm-emitting QCLs, laser action starts close to resonance (i.e., at 43.2 kV/cm vs. 45.3 kV/cm). Similarly, as for the 4.9 μm-emitting QCLs, there is strong extraction from the *ll* level, state 3, in that the 3-3′ splitting at resonance is 16.7 meV and it occurs close to threshold (i.e., at 42 kV/cm vs. 43.2 kV/cm). There is also strong extraction for state 2 via sequential coupling to: (a) extractor state 2″ (12.4 meV splitting at 39.7 kV/cm) and (b) extractor state 2′ (6.6 meV splitting at 45.3 kV/cm). Thus, the device has miniband-type extraction which, together with a 0.13 ps *ll*-level lifetime, ensures quick gain recovery. At threshold, the *ul* level is state 4, unlike for the 4.9 um-emitting QCL. At resonance, there is lasing from both the *g*_3_ and 4 states, and above resonance lasing resumes solely from state 4. We attribute this behavior to relatively weak PICT action.

The NEGF analysis shows nonthermal population as expected, in that the electron temperatures increase with increasing state energy. The most relevant electron temperatures at threshold, *T*_e,th_, are for states *g*_3_ and 4: 432 K and 500 K, respectively. As seen below, these high *T*_e,th_ values cause the carrier leakage to be dominated by leakage from states 4 and *g*_3_. We also note that the electron temperature in the *ul* level is comparable to that found via NEGF analysis of a 8.5 μm-emitting QCL (i.e., 512 K at ∼2 × *J*_th_) [3], albeit for a device that had PICT action only as far as photon-driven transport in the optical-transition region [13] due to intentional weak coupling between the injector state and the *ul* level, to allow for wavelength tunability [32].

The *ul*-level lifetime is 1.1 ps which, together with the 0.13 ps *ll*-level lifetime, provides an *η*_*tr*_ value of 89 %. For the ideal case of complete carrier-leakage suppression and unity tunneling injection efficiency, the *η*_*tr*_ value (i.e., 89 %) represents the upper limit for *η*_*i*_. By contrast, the experimentally obtained *η*_*i*_ value was only 66 % which, as we shall see below, is primarily due to strong carrier leakage.

### 3.2 Carrier-leakage analysis

**Figure 8:**
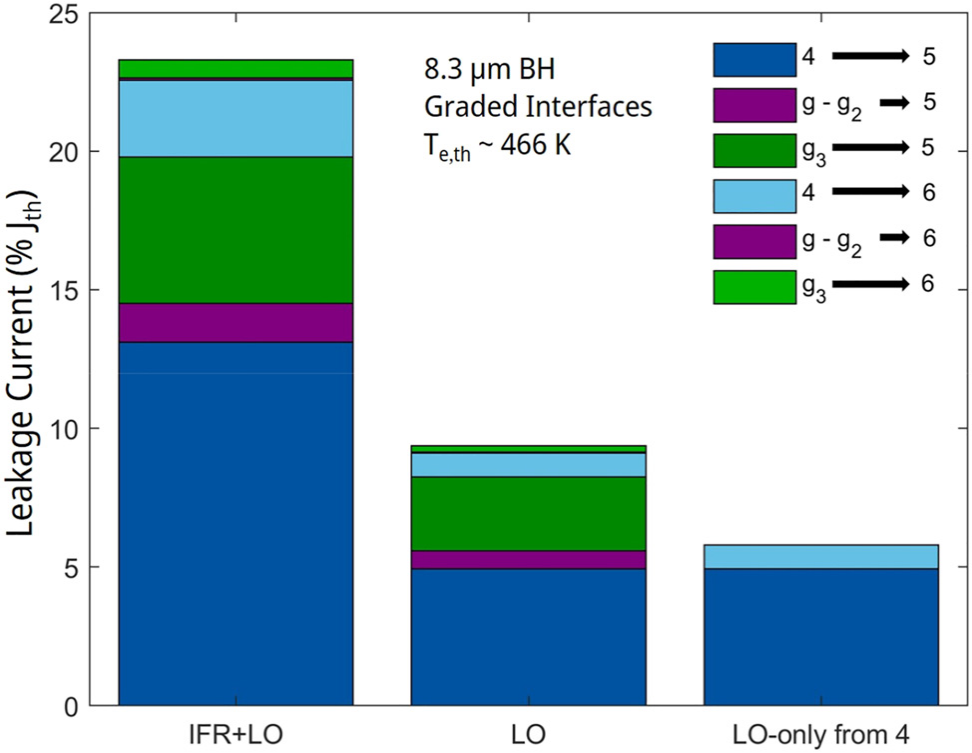
Bar graphs of the components of the relative leakage-current density through the active-region energy states 5 and 6, for the 8.3 μm-emitting QCL [6]. LO, and LO-only from 4 stand for leakage triggered only by LO-phonon scattering in the presence of elastic scattering, and leakage only from state 4, in the absence of elastic scattering, respectively.

The analysis is done, just as for the 4.9 μm-emitting QCL, by using the comprehensive carrier-leakage model from [14] with relevant-states’ sheet-carrier densities and electron temperatures provided by NEGF analysis, and using IFR- and AD-scattering rates for graded interfaces. Figure 8 shows the total *J*_*leak*_ value normalized to *J*_*th*_; the total normalized $J_{\mathrm{leak}}^{LO}$ value; and the total normalized $J_{\mathrm{leak}}^{LO}$ value in case of inelastic scattering from only the *ul* level, state 4.

The total normalized leakage, *J*_*leak*_/*J*_*th*_, reaches a value of 23.3 %, which is due to the high *T*_e,th_ values in states *g*_3_ and 4, and it occurs mostly through state 5 (i.e., a total leakage of ∼20 %). The latter is due to the relatively low value of 62 meV for the energy difference between the state 5 and the *ul* level, state 4, *E*_54_, typical of conventional 8–9 μm-emitting QCLs [23]. The leakage is triggered mostly from states 4 and *g*_3_, since they not only have the highest *T*_e,th_ values but also the strongest wavefunction overlaps with the state-5 wavefunction. Just as for the 4.9 μm-emitting QCL, the leakage is in large part IFR triggered.

Like the 4.9 μm-emitting PICT-action device, the record front-facet WPE value for long-wave infrared QCLs (i.e., 17 %), although achieved from a device possessing PICT action, proves that there is significant room for improvement, given that the WPE fundamental upper limit for ∼8 μm-emitting QCLs is ∼25 % [16]. The improvement can be achieved by designing STA-type QCL structures with PICT action *and* virtually complete carrier-leakage suppression. By employing our NEGF-based model with graded interfaces, we have obtained a preliminary STA-QCL 8.1 μm-emitting design of significant carrier-leakage suppression and, in turn, a maximum WPE value close to the fundamental limit.

### 3.3 Sensitivity analysis to variations in IFR parameters values

While keeping the Λ value constant at 6 nm, sensitivity analyses to variations in the *L*/Δ_⊥_ value and in the in Δ value on device performance reveal that the slope efficiency, *η*_*sl*_, is the most sensitive device characteristic (see Supplementary Material, section B). Maximum errors of +/−4 % in the *η*_*sl*_ value have been chosen as the criterion for a reasonably good fit to experimental data. The *η*_*sl*_ value increases with increasing *L*/Δ_⊥_ value because of less IFR-triggered carrier leakage [7]. The *η*_*sl*_ value decreases with increasing Δ value because of increased IFR-triggered carrier leakage [14]. Details of the analysis are presented in Supplementary Material, section B.

### 3.4 Comparisons to results obtained with extracted abrupt-interface IFR parameters

Using Λ = 9 nm and Δ = 0.10 nm [3], the *J*_th_ value increases from 1.37 kA/cm^2^ to 1.98 kA/cm^2^, since lasing starts at a higher field: 46.6 kV/cm. There is PICT action, but it is weaker than for the graded-interface case, as evidenced by a higher *R*_diff_ value: 1.9 Ω and a lower *J*_max_ value: 5.2 kA/cm^2^ (see Supplementary Material, section D).

## 4 Design of 8.1 μm-emitting STA-QCL with significantly enhanced wall-plug efficiency

By using basically the same structure employed for the 8.3 μm-emitting QCL [6] and of the same nominal sheet-doping density, we replaced the third barrier in the AR with an AlAs barrier. Thus, the new structure is of the STA type. That is, the barriers are stepwise tapered in the AR, which brings about two advantages as far as carrier-leakage suppression [9]: increased *E*_*5,ul*_ value, and decreased overlap of the wavefunctions for the *ul* level and the next higher AR energy level, state 5. The same graded-interfaces’ IFR parameters we found above for the 8.3 μm-emitting QCL are used, except that for the AlAs barrier we choose Δ = 0.17 nm, to reflect what we found for the 4.9 μm-emitting QCL grown by the same method (i.e., GS-MBE).

### 4.1 NEGF-based analysis

**Figure 9:**
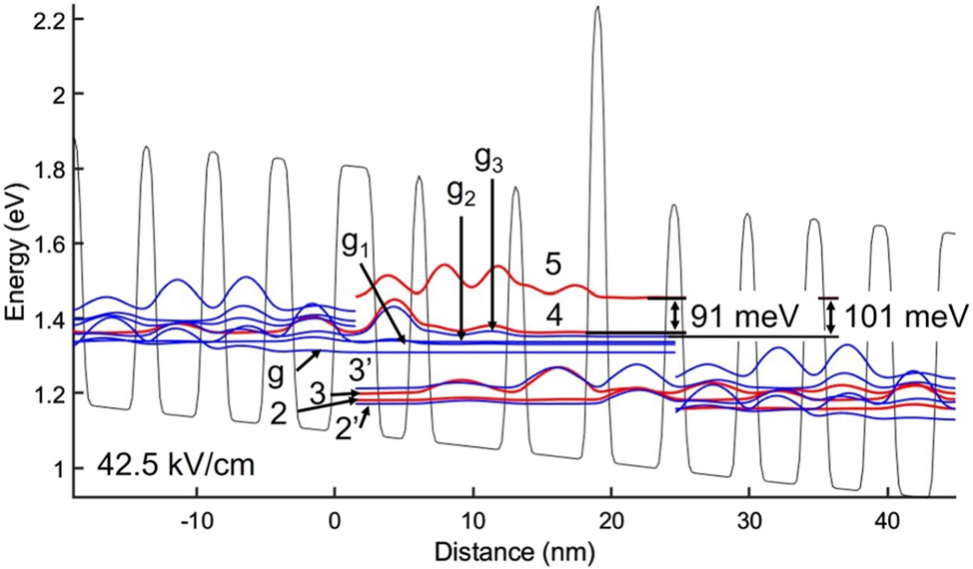
Conduction-band diagram and relevant wavefunctions for 8.1 μm-emitting STA-type QCL, at threshold. *g*_3_ is the upper laser level.

The CB diagram and relevant wavefunctions are shown in Figure 9. Notably, the *E*_54_ value has increased from 62 meV to 91 meV. However, at threshold, the lasing transition occurs from state *g*_3_, since injection from the prior-stage state 2 occurs into it; thus, the relevant energy difference is *E*_5,g3_, which is 101 meV. In this case, lasing threshold occurs right at the resonances between the prior-stage states 2 and 2′, and between states *g*_3_ and state 4, respectively (i.e., at 42.5 kV/cm), as clearly evidenced by the virtual complete overlap of their respective wavefunctions. The splitting energy at resonance is 10.5 meV; thus, there is strong coupling. In addition, the device has a more diagonal lasing transition than the 8.3 μm-emitting QCL (i.e., the *z*_ul,ll_ value decreases from 16.4 Å to 9.2 Å). Thus, one expects stronger PICT action.

NEGF analysis provides nonthermal population, with *T*_e,th_ values for states *g*_3_ and 4: 455 K and 499 K, respectively. Due to the increased lasing-transition diagonality, the *ul*-level lifetime is 2.5 ps compared to 1.1 ps for the 8.3 μm-emitting device. Together with a 0.13 ps *ll*-level lifetime, the *η*_*tr*_ value is 94.8 %.

**Figure 10:**
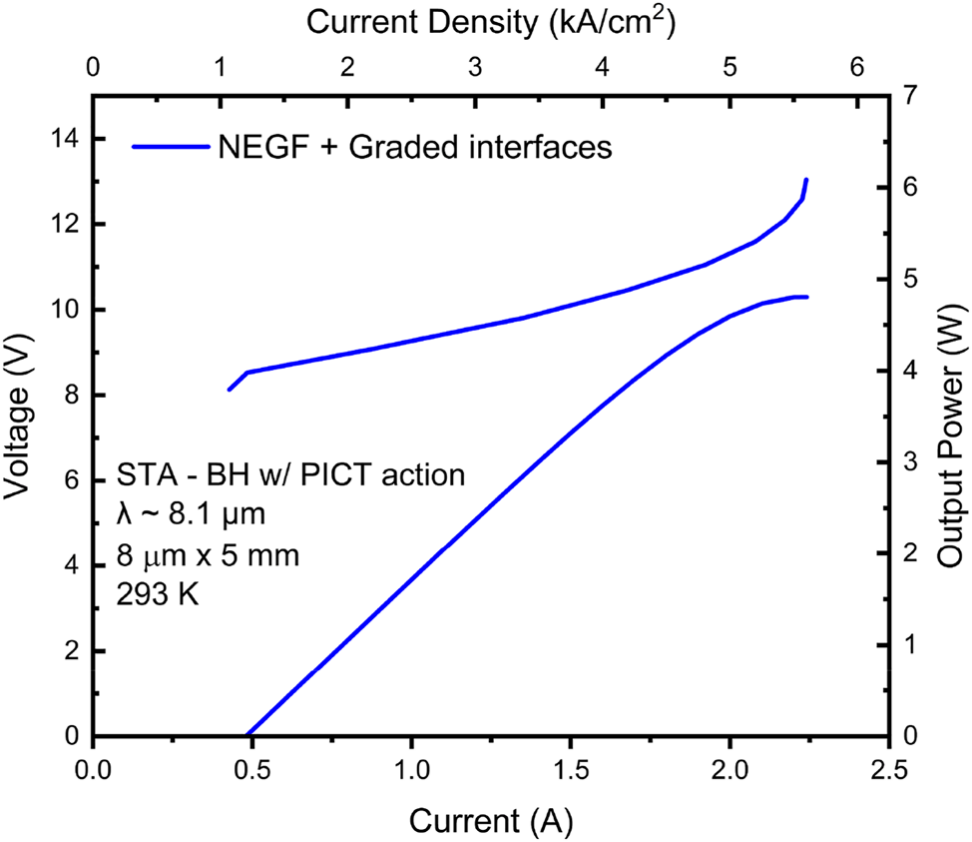
Voltage– and light–current curves for 8.1 μm-emitting STA-type QCL with photon-induced carrier transport, calculated using the model with graded interfaces. The employed graded-interface IFR-scattering parameters are: Δ = 0.11 nm and 0.17 nm for all interfaces except those at the AlAs barrier, and for the interfaces bounding the AlAs barrier, respectively; Λ = 6 nm; *L* = 0.4 nm; and Δ_⊥_ = 0.1 nm.

Figure 10 shows the generated V–I and L–I curves for a device of the same pumped area, waveguide and mirror losses as for the 8.3 μm-emitting QCL. The stronger PICT action is evidenced by a lower calculated *R*_diff_ value: 1.5 Ω versus 1.65 Ω, and by a higher *J*_max_ value: 5.6 kA/cm^2^ vs. 5.45 kA/cm^2^. Now the *J*_max_ value is ∼20 % higher than for conventional ∼8 μm-emitting QCLs of the same injector doping level. The maximum peak power is 4.8 W compared to 4.2 W for the 8.3 μm-emitting QCL.

### 4.2 Carrier-leakage analysis

**Figure 11:**
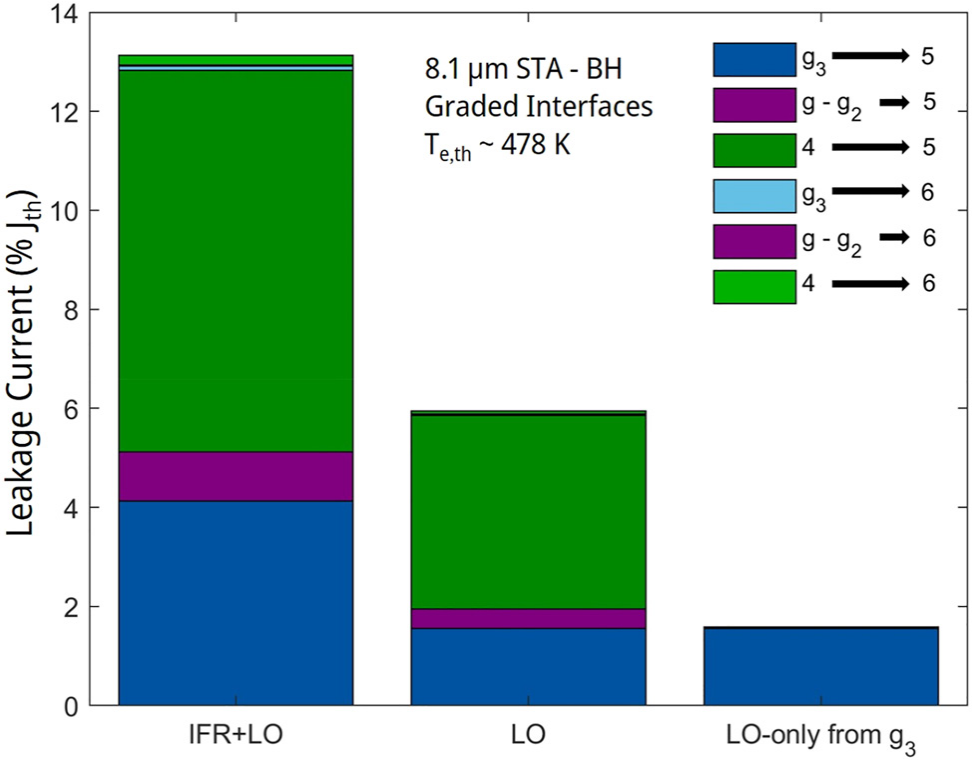
Bar graphs of the components of the relative leakage-current density, at threshold, through the active-region energy states 5 and 6, for the 8.1 μm-emitting STA-type QCL. LO, and LO-only from *g*_3_ stand for leakage triggered only by LO-phonon scattering in the presence of elastic scattering, and leakage only from state *g*_3_ in the absence of elastic scattering, respectively.

As mentioned above, we use the carrier-leakage model from [14] with energy-states’ sheet-carrier densities and electron temperatures provided by NEGF analysis, and IFR- and AD-scattering rates for graded interfaces. The results are shown in Figure 11.

The total normalized leakage, *J*_*leak*_/*J*_*th*_, reaches a value of 13.1 %; that is, ∼56 % the value for the 8.3 μm-emitting QCL. The *η*_inj_ value is 84.3 % which, together with the *η*_*tr*_ value, results in an internal efficiency *η*_*i*_ value of 79.9 %. This is a significant improvement over the 66 % value obtained for the 8.3 μm-emitting devices [6]. The higher *η*_*i*_ value is reflected in a significant increase in slope efficiency; that is, 3.29 W/A (Figure 10) versus 2.6 W/A [6]. Similarly, the decrease in carrier leakage is reflected in a lower *J*_th_ value: 1.21 kA/cm^2^ vs. 1.37 kA/cm^2^ [6]. This means that the absolute leakage-current density has dropped to 0.16 kA/cm^2^ from 0.33 kA/cm^2^; i.e., it has basically halved. It should be pointed out that this is a preliminary STA design, in that optimized STA designs can reach *J*_*leak*_/*J*_*th*_ values as low as 5 % [14].

### 4.3 Wall-plug efficiency

**Figure 12:**
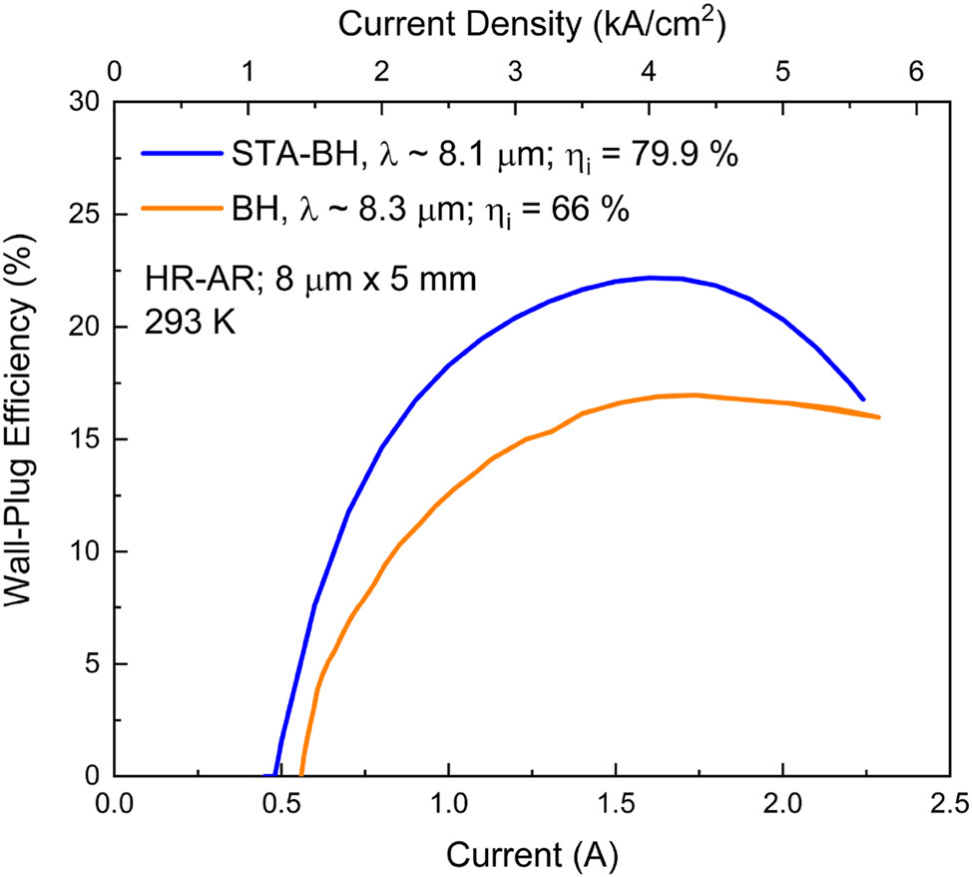
Wall-plug efficiency vs. drive current curves for the published 8.3 μm-emitting QCL [6] and the designed 8.1 μm-emitting STA-type QCL. *η*_*i*_ is the internal efficiency [4].

The combined effect of suppressed carrier leakage and stronger PICT action provides a front-facet maximum WPE value of 22.2 %, compared to the current record of 17 % (see Figure 12). Thus, a value close to the fundamental limit of ∼25 % [16] is achieved.

## 5 Conclusions

Modeling with graded interfaces accurately reproduces the characteristics of record-high performance mid- and long-wave infrared QCLs. The key features for maximizing the wall-plug efficiency are direct injection from a prior-stage, low-energy (active-region) state into the upper laser level, and strong photon-induced carrier transport. That is, unlike conventional QCLs, carrier injection occurs at the exit barrier of the prior stage into the upper laser level via coherent and incoherent tunneling from a low-energy active-region state.

We also find that, despite record wall-plug efficiency values: 27 % at *λ* ∼ 4.9 μm and 17 % at *λ* ∼ 8.3 μm, there is significant normalized leakage-current density: 26–28 % and 23.3 %, respectively, due in large part to high average electron temperature in the upper laser level and an energy state adjacent to it: 1060 K and 466 K, respectively. That means there is significant room for improvement via carrier-leakage suppression; thus, potential to approach the fundamental upper limits in wall-plug efficiency: 40 % at *λ* ∼ 4.9 μm and 25 % at *λ* ∼ 8.3 μm. Given its accuracy, modeling with graded interfaces becomes the design tool for reaching those performance goals. We have used this new QCL-design tool for obtaining a preliminary design for a 8.1 μm-emitting QCL with significant carrier-leakage suppression, which reaches a maximum wall-plug efficiency of 22.2 %, thus, close to the ∼25 % upper limit.

## Supplementary Material

Supplementary Material Details
