# Supplementary material for: Modeling with graded interfaces: Tool for understanding and designing record-high power and efficiency mid-infrared quantum cascade lasers
Source: Nanophotonics. 2024 Jan 12;13(10):1745–57. doi: 10.1515/nanoph-2023-0687 (PMC11614349; doi:10.1515/nanoph-2023-0687)
Supplement: Supplementary file 1 — Supplementary Material Details [file j_nanoph-2023-0687_suppl_001.pdf]

Supplementary Material

Modeling with Graded Interfaces: Tool for Understanding and Designing Record-High Power and Efficiency Mid-Infrared Quantum Cascade Lasers

S. Suri [1], B. Knipfer[2], T. Grange[3] , H. Guo[1], J. D. Kirch[1], L. J. Mawst[1], R A. Marsland[2], and D. Botez[1]

[1]Dept. of Electrical and Computer Engineering, Univ. of Wisconsin-Madison, Madison, WI 53706, USA

[2]Intraband, LLC, Madison, WI 53726, USA, [3]nextnano Lab, 12 chemin des prunelles, 38700 Corenc, France

botez@.engr.wisc.edu

A. Experimental data from 4.6 μm-emitting QCL designed with the graded-interfaces model

We performed a preliminary design for a 4.6 μm-emitting STA-type [1] QCL with basically the same IFR parameters found via APT [2]: Δ = 0.14 nm at moderately strained AlInAs-barrier interfaces, and 0.2 nm at highly strained (i.e., AlAs) barrier interfaces; Λ = 6 nm; and L = 0.55 nm. In addition, a value of 0.1 nm was assumed for $\Delta_{\perp}$. MOCVD-grown wafers, with a nominal injector doping: n_s = 1.04x10^{11}cm^{-2}, were processed into 20 μm-wide ridges with HR-coated rear facets and provided the following electro-optical characteristics: J_{th} = 1.25 kA/cm^2; slope efficiency, η_{sl} = 4.65 W/A; R_{diff} = 1.3 Ω, and a maximum front-facet wall-plug efficiency, $\eta_{wp,max}$ = 17.6 %. By comparison, the values predicted by the model were: J_{th} = 1.24 kA/cm^2; η_{sl} = 4.27 W/A; R_{diff} = 1.34 Ω, and $\eta_{wp,max}$ = 18 %. Comparisons of experimental data vs. predicted characteristics are shown in Figs. S1 (a) and (b).

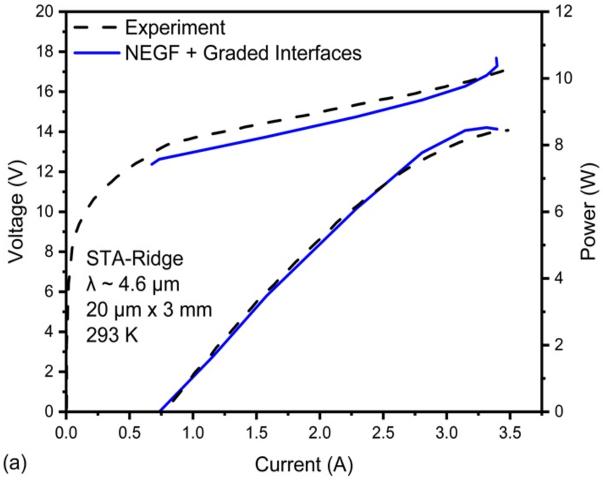

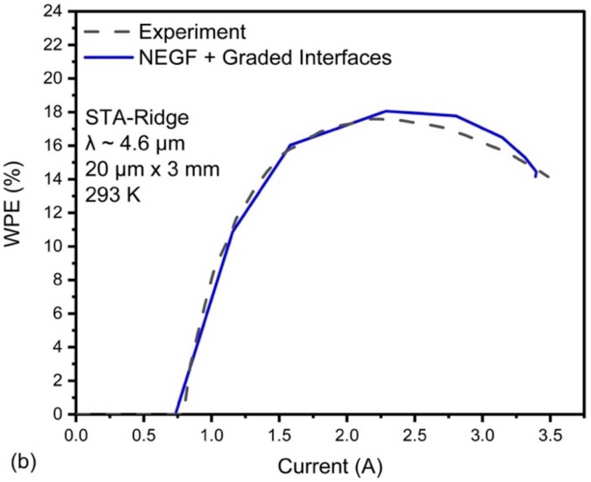

Figure S1: Comparison of experimental data from ridge-guide STA-type QCL modeled with graded interfaces vs. calculated results: (a) L-I-V curves; (b) Wall-plug efficiency vs. I curves.

As can be seen, there is good agreement between experiment and theory. The experimental V-I curve displays strong PICT action, in that R_{diff} = 1.3 Ω; that is, 55 % of that for conventional QCLs of same pumped area (i.e., 2.33 Ω), and a J_{max} value of 5.85 kA/cm^2; that is, 1.35 times higher than that for conventional 4.6 μm-emitting QCLs of same injector doping level (i.e., 4.35 kA/cm^2). These comparisons confirm the accuracy of both the graded-interfaces model and the IFR parameters obtained from analysis of APT results.

In addition, we had material from the same wafer processed into narrow (6 μm) buried-heterostructure (BH), 5 mm-long chips with HR-coated rear facets and 10 %-coated front facets. The narrow buried ridge was used for obtaining single-spatial-mode operation. Experimental results are shown in Fig. S2. A front-facet $\eta_{wp,max}$ value of 19.1 % is obtained, which is higher than

the best front-facet results reported to date from vertical-transition (i.e., no PICT action) 4.6-5.0 μm-emitting QCLs (i.e., 15.1 % [3] and 14 % [4]). However, the result is lower than the record value obtained from the 4.9 μm-emitting QCL [5]. We attribute the difference to two issues: (a) this preliminary design has two parasitic, hot states above the *ul* level; thus, the calculated relative carrier-leakage density, $J_{\mathrm{leak}}/J_{\mathrm{th}}$, at threshold, is rather high: ~ 30 %; (b) the crystal-growth conditions have not yet been optimized for a low waveguide loss, α_{w} (i.e., ~ 0.5 cm^{-1} as achieved for the devices reported in [3],[6], and in [5]). With further design optimization for low relative leakage (i.e., ≤ 10 %), and optimization of the crystal-growth conditions for lowering the α_{w} value we are confident that the $\eta_{\mathrm{wp,max}}$ value will significantly increase. For instance, for a more recent less leaky design (i.e., 17 % relative leakage), and using the IFR parameters we found for the 4.9 μm-emitting QCL [5], we project that a BH device of same dimensions as in [5] will lead to a front-facet $\eta_{\mathrm{wp,max}}$ value of 31.2 %; that is, already higher than the current record (i.e., 27 %).

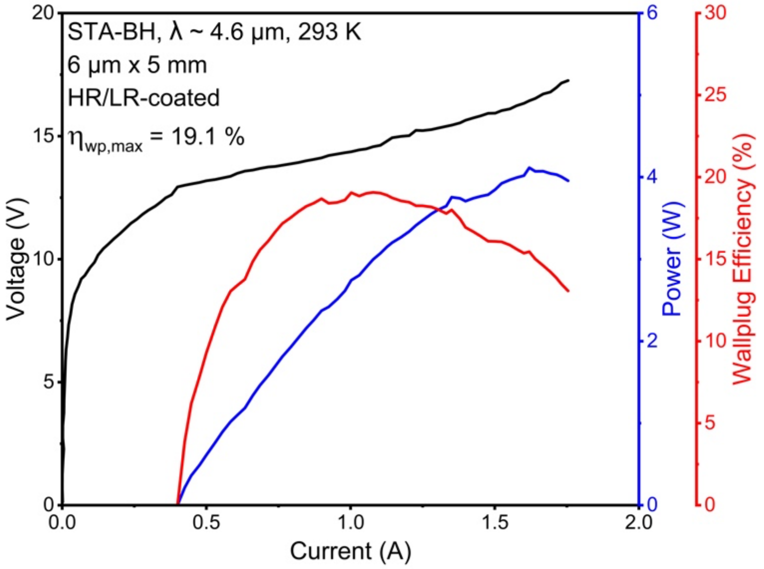

Fig. S2: L-I-V and WPE-I curves for BH-type, HR/LR-coated laser fabricated from the STA-type QCL material of preliminary PICT-action design.

B. Sensitivity analysis to variations in IFR parameters on the performance of the published 4.9 μm- and 8.3 μm-emitting QCLs

The in-plane correlation length Λ is kept constant at 6 nm, as per its experimental determination from APT-results analysis of a 4.6 μm-emitting STA-type QCL structure [2]. It is also worth noting that the Δ value of ~ 0.135 nm, measured [2] for moderately strained AlInAs/InGaAs interfaces, justifies the selection of a value of 0.13 nm for virtually identical moderately strained AlInAs/InGaAs interfaces of the studied 4.9 μm-emitting QCL structure. That leaves as the only IFR parameters which have been extracted by matching the J_{th} and L-I-V curves: the graded-interfaces' width, L, and the axial correlation length, $\Delta_{\perp}$. However, the scattering-rate reduction factor due to graded interfaces [7], *F*, is the only function of those parameters, and, furthermore, is a function of their ratio. Thus, the analysis is done as a function of variations in the $L/\Delta_{\perp}$ value.

We have found that the slope efficiency, η_{sl}, is the most sensitive parameter to variations in $L/\Delta_{\perp}$ and Δ values. We set as a tolerable variation in the η_{sl} value: +/- 4 %.

B.1 Analysis of device performance to variations in the L/Δ⊥ value

B.1.1 4.9 µm-emitting QCL

Since this device was found to operate in a dominant gain peak at a drive level ~ 1.4 x J_{th}, corresponding to lasing transitions from state g_4 to the lower-laser (ll) levels 3 and 3', as well as to the resonance field between states g_4 and 4, calculations are done at the g_4/4 resonance points for each L/Δ⊥ value. The results are summarized in Tables S1 and S2.

Table S1: Changes in η_{sl} with varying the L/Δ⊥ value.

L/Δ⊥	× J_{th}	λ (µm)	J_{max} (kA/cm²)	η_{sl} (W/A)	η_{tr} (%)	J_{leak}/J (%)	η_p (%)	η_{sl} x η_p/η_p (W/A)	Error (%)
3	1.3	4.7	5.65	5.47	97	32.6	67	5.35	- 2.2
**3.5**	1.5	4.72	5.75	**5.56**	96.9	31.1	**69**	5.47	- **1.6**
**4**	1.4	4.8	5.76	**5.72**	97	28	**72**		
**4.5**	1.5	4.8	5.83	**5.94**	96.5	24.7	**75**	5.98	+ **0.7**
5	1.6	4.84	5.9	5.96	96	23.2	77	6.1	+ 2.3

First, the results show that the η_{sl} value varies within +/- 4 % with respect to the value found to match experiment (i.e., 5.72 W/A for L/Δ⊥ = 4); that is, the η_{sl} value is: 5.72 W/A $^{+3.8\,\%}_{-2.8\,\%}$, for the range in L/Δ⊥: 4 $^{+0.5}_{-0.5}$. The reason behind the η_{sl} variations are variations in the differential pumping efficiency $\eta_p = 1 - (J_{leak}\,/\,J)$, since $\eta_{sl} \propto \eta_{tr}\eta_p$ [4], while the lasing-transition efficiency, η_{tr}, hardly varies with L/Δ⊥ . This happens because variations in the IFR components of both the global upper-laser (ul)- and ll-level lifetimes, $\tau_{ul,g}$ and $\tau_{ll,g}$, cause the ratio $\tau_{ll,g}/\tau_{ul,g}$ to hardly vary. Also shown in the table is a comparison of scaled η_{sl} values with η_p with respect to the L/Δ⊥ = 4 case. As can be seen, the errors are ≤ 1.6 % over the acceptable range in L/Δ⊥ (i.e., 4 $^{+0.5}_{-0.5}$), and ≤ 2.3 % over the entire L/Δ⊥ range. We conclude that $\eta_{sl} \propto \eta_p$; i.e., that η_{sl} variations are due to relative carrier-leakage variations. Why this happens is evident from Table S2 and its discussion.

Table S2: Changes in J_{leak}^{IFR} with varying the F factor.

L/Δ⊥	J_{leak}^{IFR}/J (%)	J_{leak}^{IFR} (kA/cm²)	F	J_{leak}^{IFR}x F/F (kA/cm²)	Error (%)
3	27.5	0.46	0.456	0.52	+ 13
**3.5**	26.1	**0.52**	**0.414**	0.47	- **9.6**
**4**	23.4	**0.43**	**0.378**		
**4.5**	20.1	**0.42**	**0.348**	0.396	- **5.7**
5	18.7	0.39	0.321	0.365	- 6.4

We established that $\eta_{sl} \propto \eta_p$. The η_p term varies with changes in the $J_{leak}\,/\,J$ ratio, but only the IFR part of J_{leak}, J_{leak}^{IFR}, varies with L/Δ⊥. We find that the percentage of J_{leak} that corresponds to J_{leak}^{IFR} is basically constant over the 4 $^{+0.5}_{-0.5}$ range in L/Δ⊥: 84 $^{-2}_{+0}$ %, so its impact on the η_{sl} value is the same over the 4 $^{+0.5}_{-0.5}$ range. Next, let us look at the expression for J_{leak}^{IFR} [8]:

$$J_{leak,ul,5}^{IFR} = \frac{en_{ul}}{\tau_{5,ul}^{IFR}} \frac{\tau_{5,tot}^{LO,IFR,AD}}{\tau_{5,leak}^{LO,IFR,AD}} I_{nm} \left(\frac{E_{5,ul}}{kT_{e,ul}} \right) \exp\left(-\frac{E_{5,ul}}{kT_{e,ul}} \right) \tag{1a}$$

where we consider IFR-triggered leakage from the ul level (i.e., g_4) through the active-region state 5. The IFR backscattering rate from state 5 to state g_4 is obtained from Eq. (3) in the text.:

$$\frac{1}{\tau_{5,g4}^{IFR}} \cong \frac{\pi}{\hbar^3}\Lambda^2 F \sum_i m_{ci}\, \Delta_i^2 \delta V_i^2 \varphi_5^2(z_i)\varphi_{g4}^2(z_i) exp\left(-\frac{\Lambda^2 m_{ci} E_{5,g4}}{2\hbar^2}\right) \quad (1b)$$

Over the 4 $^{+0.5}_{-0.5}$ range: (a) the interface overlap factor (IOF) [8]: $\sum_i \varphi_5^2\,(z_i)\varphi_{g4}^2(z_i)$, value is found to vary little: $1.78^{+0.09}_{-0.09} \times 10^{-4}$, and (b) the $E_{5,g4}$ value varies negligibly: 119^{+1}_{-1} meV. Therefore, we expect $1\,/\,\tau_{5,g4}^{IFR}$ to basically be proportional with the *F* factor. Looking next at Eq. (1a) we find that: (a) n_{ul} varies negligibly: $5.66\ ^{-0.02}_{+0.02}\ \times 10^9 cm^{-2}$; (b) the lifetime ratio, which is the percentage of the carriers excited to state 5 which relax to all lower-AR and extractor states (i.e., leaked carriers out of excited carriers), also varies negligibly: 26 $^{+0.7}_{-0.7}$ %; (c) the $E_{5,g4}/T_{e,g4}$ value varies as such: $0.118\ ^{-0.003}_{-0.010}$ meV/K. The term $I_{5,g4}$ has been found [9] to be a weak function of both temperature and excitation energy, $E_{5,g4}$. Therefore, since $1\,/\,\tau_{5,g4}^{IFR}$ is proportional with the *F* factor, one expects $J_{leak,g4,5}^{IFR}$ to basically be proportional with *F*. Also shown in the table is a comparison of scaled values of $J_{leak,g4,5}^{IFR}$ with *F* with respect to the *F* value for the L/$\Delta_\perp$ = 4 case. The errors with respect to calculated values are: - 5.7 % and - 9.6 %. Therefore, $J_{leak,g4,5}^{IFR}$ is reasonably proportional with *F*. Much smaller errors are expected if the drive currents were identical, as we shall see below for the 8.3 μm-emitting QCL, for which all calculations are performed at the same drive level; that is, at threshold.

Given $\eta_{sl} \propto \eta_p$, since $\eta_p = 1 - (J_{leak}\,/\,J)$ and J_{leak}^{IFR} is ≅ 84 % of J_{leak}, the η_{sl} value variations (i.e., 5.72 W/A $^{+3.8\ \%}_{-2.8\ \%}$) can be directly attributed to variations in the *F* factor. In turn, the *F* factor is inversely proportional with L/$\Delta_\perp$ (Fig. S3), as expected since wider graded regions lead to reductions in the IFR scattering rate [7]. In short, since an increase in L/$\Delta_\perp$ leads to less IFR-triggered carrier leakage, the η_{sl} value increases with increasing L/$\Delta_\perp$ value. This explains the η_{sl} increase with L/$\Delta_\perp$ in Table S1.

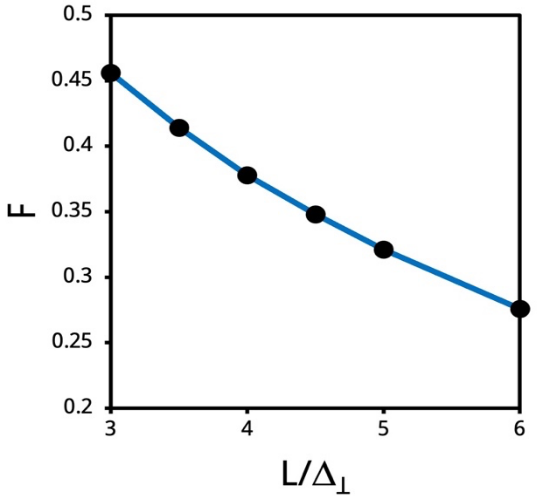

Fig. S3: The scattering-rate reduction factor vs. the L/$\Delta_\perp$ ratio.

The other changes over the 4 $^{+0.5}_{-0.5}$ range in L/$\Delta_\perp$ are small. For J_{max}: 5.76 $^{+0.07}_{-0.01}$ kA/cm^2, which corresponds to variations of only +1.2 % and -0.2 %, since the coupling between the injecting states 2 and 2', and the g_4 and 4 states stays basically the same: 7.5 meV. We don't expect L to be

wider than 0.55 nm or narrower than 0.30 nm. Thus, for the L/$\Delta_{\perp}$ value that best fits experimental data (i.e., 4) the $\Delta_{\perp}$ value may well be in the 0.08-0.14 nm range.

B.1.2 8.3 µm-emitting QCL

This device was found to operate in a dominant gain peak at a threshold, corresponding to lasing transitions from state 4 to the *ll* levels 3 and 3'. The resonance is at 2.3 kV/cm above threshold, where there are comparable gain peaks for transitions from both states 4 and g_3, and then above resonance lasing from state 4 returns to be the dominant one. This behavior reflects initial injection from state 2 of the prior stage into the *ul* level, state 4, as opposed to injection from state 2 into the *ul* level, state g_3, for the designed 8.1 µm-emitting QCL. The results are summarized in Tables S3 and S4.

Table S3: Changes in η_{sl} with varying the L/$\Delta_{\perp}$ value.

L/$\Delta_{\perp}$	J_{th} (kA/cm^2)	J_{max} (kA/cm^2)	η_{sl} (W/A)	η_{tr} (%)	J_{leak}/J_{th}	η_p (%)	η_{sl} x η_p/η_p (W/A)	Error (%)
**3**	1.39	5.32	**2.53**	89.2	26.2	**73.8**	2.51	**- 0.8**
**3.5**	1.37	5.46	**2.57**	89	24.6	**75.4**	2.57	**0**
**4**	1.37	5.45	**2.61**	88.9	23.3	**76.7**		
**4.5**	1.37	5.45	**2.65**	88.7	22	**78.0**	2.65	**0**
**5**	1.37	5.43	**2.67**	88.7	21.2	**79.0**	2.69	**+ 0.7**
6	1.34	5.4	2.8	88	17.2	82.8	2.82	+ 0.7

First, the results show that the η_{sl} value varies within +/- 4 % with respect to the value found to match experiment (i.e., 2.61 W/A for L/$\Delta_{\perp}$ = 4). More specifically, the η_{sl} value is: 2.61 W/A $^{+2.3\,\%}_{-3.1\,\%}$ for the range in L/$\Delta_{\perp}$: 4 $^{+1}_{-1}$. The reason behind the η_{sl} variations are variations in the differential pumping efficiency $\eta_p = 1 - (J_{leak}\,/\,J)$, since $\eta_{sl} \propto \eta_{tr}\eta_p$, while the lasing-transition efficiency η_{tr} hardly varies with L/$\Delta_{\perp}$, just like for the 4.9 µm-emitting device. As can be seen, the errors are ≤ 0.8 % over both the acceptable range in L/$\Delta_{\perp}$ (i.e., 4 $^{+1}_{-1}$) and over the entire L/$\Delta_{\perp}$ range. We conclude that $\eta_{sl} \propto \eta_p$; i.e., that η_{sl} variations are due to relative carrier-leakage variations. Why does this happen is evident from Table S4 and its discussion.

Table S4: Changes in J_{leak}^{IFR} with varying the *F* factor.

L/$\Delta_{\perp}$	J_{leak}^{IFR}/J_{th} (%)	J_{leak}^{IFR} (kA/cm^2)	*F*	J_{leak}^{IFR} x F/F (kA/cm^2)	Error (%)
**3**	16.5	**0.23**	**0.456**	0.23	**0**
**3.5**	15.1	**0.21**	**0.414**	0.21	**0**
**4**	13.93	**0.19**	**0.378**		
**4.5**	12.86	**0.176**	**0.348**	0.175	- **0.6**
**5**	12.08	**0.165**	**0.321**	0.16	**- 2.4**
6	9.33	0.125	0.276	0.14	+ 12

As for the 4.9 µm-emitting device, the table shows a comparison of scaled values of $J_{leak,45}^{IFR}$ with *F* with respect to the *F* value for the L/$\Delta_{\perp}$ = 4 case. The errors with respect to calculated values are: - 0 % and - 2.4 %; that is, significantly much lower than for the 4.9 µm-emitting device, since that comparison was done for data at somewhat different drive levels above threshold, while

this is done at threshold [As seem from Table S3, J_{th} is basically the same over the acceptable range in L/Δ⊥ (i.e., 4^{+1}_{-1})]. Therefore, the $J^{IFR}_{leak,g4,5}$ value is very accurately proportional with *F*.

Given $\eta_{sl} \propto \eta_p$, since $\eta_p = 1 - (J_{leak} \,/\, J)$ and we found that J^{IFR}_{leak}/J_{th} is 60^{-3}_{+3} % of J_{leak}/J_{th}, the η_{sl} value variations (i.e., 2.61 W/A $^{+2.3\,\%}_{-3.1\,\%}$) can be directly attributed to variations in the *F* factor. Then, we reach the same conclusion as for the 4.9 μm-emitting device: the η_{sl} value increases with increasing L/Δ⊥ value because less IFR-triggered carrier leakage is associated with increasing L/Δ⊥ value. As for J_{max}, just as for the 4.9 μm-emitting QCL, the variations are small: 5.45 kA/cm^2 $^{-0.4\%}_{-2.4\%}$.

As mentioned above, we don't expect L to be wider than 0.55 nm or narrower than 0.30 nm. Thus, just as for the 4.9 μm-emitting device, for the L/Δ⊥ value that best fits the experimental data (i.e., 4) the Δ⊥ value may well be in the 0.08-0.14 nm range.

B.2 Analysis of device performance to variations in the Δ value

B.2.1 4.9 μm-emitting QCL

B.2.1.1 Varying-Δ device vs. uniform-Δ device

The device that matched experimental data has Δ values of 0.10 nm, 0.13 nm and 0.17 nm for the interfaces of the short lattice-matched barrier, moderately strained barriers, and the tall heavily strained exit barrier, respectively. Table S5 shows a comparison to a device of same Δ value: 0.13 nm, for the interfaces of all barriers.

Table S5: Changes in η_{sl} for the varying-Δ case vs. the uniform-Δ case.

Δ (nm)	× J_{th}	λ (μm)	J_{max} (kA/cm^2)	η_{sl} (W/A)	η_{tr} (%)	J_{leak}/J (%)	η_p (%)	η_{sl} x η_p/η_p (W/A)	Error (%)
0.13	1.4	4.8	5.73	**5.57**	96.9	29.8	**70.2**	5.58	+ **0.2**
**0.1-0.13-0.17**	1.4	4.8	5.76	**5.72**	97.1	27.9	**72**		

The η_{sl} value decreases with the pumping efficiency η_p (with an error of only 0.2 %) when going from the varying-Δ case to the uniform-Δ case, since the lasing-transition efficiency η_{tr} stays basically the same. This fact is somewhat counterintuitive. On the one hand, although the low-Δ, lattice-matched barrier significantly lengthens the IFR part of the *ul*-level lifetime (i.e., from 10.9 ps in the uniform-Δ case to 13.9 ps in the varying-Δ case) the effective global *ul*-level lifetime, $\tau_{up,g}$, increases slightly (from 2.18 ps to 2.28 ps) since LO and AD scattering primarily determine the *ul*-level lifetime for 4.5-5.0 μm-emitting QCLs [4]. On the other hand, the high-Δ tall exit barrier shortens the IFR part of the global *ll*-level lifetime, $\tau^{IFR}_{ll,g}$, albeit negligibly (from 0.106 ps to 0.102 ps); thus, the *ll*-level lifetime stays basically the same (0.07 ps). In turn, the ratio $\tau_{ll,g} \,/\, \tau_{up,g}$ decreases slightly, leading to a small increase in the η_{tr} value (0.2 %).

The actual impact of the low-Δ, lattice-matched barrier is to shorten the $1 \,/\, \tau^{IFR}_{5,ul}$ part of the IFR-triggered leakage current [see Eqs. 1 (a) and (b)] (i.e., from 1.05 ps^{-1} to 0.92 ps^{-1}), thus decreasing J^{IFR}_{leak} by ~ 13 %, which leads to a 2.6 % increase in η_p, the actual increase in slope efficiency. That is, going from the uniform-Δ structure to the varying-Δ structure accounts for the almost 3 % increase in the η_{sl} value, which matches experiment (i.e., 5.76 W/A).

B.2.1.2 Effect of varying the Δ value of the moderately strained barriers' interfaces

Table S6 shows a comparison between devices of three different **Δ** values: 0.12 nm, 0.13 nm and 0.14 nm, for the interfaces of moderately strained barriers.

Table S6: Changes in η_{sl} for varying Δ value of the interfaces bounding the moderately strained barriers.

Δ (nm)	× J_{th}	λ (μm)	J_{max} (kA/cm^2)	η_{sl} (W/A)	η_{tr} (%)	J_{leak}/J (%)	η_p (%)	η_{sl} x η_p/η_p (W/A)	Error (%)
0.1-**0.12**-0.17	1.5	4.8	5.83	**5.94**	96.8	25	**75**	5.96	+ **0.3**
0.1-**0.13**-0.17	1.4	4.8	5.76	**5.72**	97.1	27.9	**72**		
0.1-**0.14**-0.17	1.35	4.8	5.66	**5.5**	97.2	30.9	**69.1**	5.49	- **0.2**

First, we note that the 0.10-0.13-0.17 case matches best with the experimental values for η_{sl} and J_{max} (i.e., 5.72 W/A and ~ 5.75 kA/cm^2) [5]. The η_{sl} value varies within the +/- 4 % range with respect to the value found to match experiment. More specifically, the η_{sl} value is: 5.72 W/A $^{-3.9\,\%}_{+3.9\,\%}$ for the range in Δ for moderately strained barriers' interfaces: 0.13 $^{+0.01}_{-0.01}$ nm. These variations are due to variations in the differential pumping efficiency $\eta_p = 1 - (J_{leak} / J)$, since $\eta_{sl} \propto \eta_{tr}.\eta_p$, while the lasing-transition efficiency η_{tr} hardly varies with Δ. The latter, just as in the previous subsection, is due to the fact that the variations in Δ, while affecting the IFR part of the *ul*-level lifetime (13.9 $^{-0.11}_{+0.12}$ ps), have a negligible effect on the $\tau_{ll,g} / \tau_{up,g}$ ratio. Scaling η_{sl} values with η_p with respect to the 0.10-0.13-0.17 case gives errors ≤ 0.3 %; thus, the conclusion is that $\eta_{sl} \propto \eta_p$. The reason behind this behavior is clarified from data in Table S7.

Table S7: Changes in J_{leak}^{IFR} with varying the Δ^2 value of the moderately strained barriers' interfaces.

Δ (nm)	J_{leak}^{IFR}/J (%)	J_{leak}^{IFR} (kA/cm^2)	J_{leak}^{IFR} x Δ^2/Δ^2 (kA/cm^2)	Error (%)
0.1-**0.12**-0.17	20.13	**0.39**	0.36	- **7.7**
0.1-**0.13**-0.17	23.3	**0.42**		
0.1-**0.14**-0.17	25.65	**0.44**	0.48	+ **9**

Just as for the L/Δ$_\perp$ study, we have established that $\eta_{sl} \propto \eta_p$. Again η_p varies with changes in the J_{leak} / J ratio, but only the IFR portion of J_{leak}, J_{leak}^{IFR}, varies with Δ [see Eqs. (1a) and (1b)]. We find that the percentage of J_{leak} that corresponds to J_{leak}^{IFR} is basically constant over the range in Δ of moderately strained barriers' interfaces: 84$^{-1.0}_{-2.5}$ %, so its impact on the η_{sl} value is basically the same over the 0.12-0.14 nm range in Δ. Over the same range the IOF value, defined above, and the $E_{5,g4}$ value stay the same: 1.75 x10^{-4} and 120 meV, respectively. Looking at Eq. (1b), 1 / $\tau_{5,g4}^{IFR}$ should be reasonably proportional with Δ^2 of moderately strained barriers' interfaces, given that there are different Δ values for the short and tall barriers in the AR. Looking next to the J_{leak}^{IFR} expression [Eq. (1a)], we find, just as for the L/Δ$_\perp$ study, that all parameters, except 1 / $\tau_{5,g4}^{IFR}$, vary negligibly; thus, we expect J_{leak}^{IFR} to be reasonably proportional with Δ^2. Scaling the J_{leak}^{IFR} value for the 0.10-0.13-0.17 case with Δ^2, we find from Table S7 that the errors are reasonably low: +9 % and – 7.7 % (We'll find below that for the 8.3 μm-emitting QCL, which has a uniform Δ value, the errors are approximately half those found here).

Given $\eta_{sl} \propto \eta_p$, since $\eta_p = 1 - (J_{leak} / J)$ and J_{leak}^{IFR} is ~ 84 % of J_{leak}, the η_{sl} value variations (i.e., 5.72 W/A $^{-3.9\,\%}_{+3.9\,\%}$) can be directly attributed to variations in the Δ value of moderately strained barriers' interfaces. In short, the higher the in-plane RMS roughness is, the more IFR-triggered leakage occurs, which explains the decrease in η_{sl} values with increasing the Δ value (of moderately strained barriers' interfaces) in Table S6.

B.2.2 8.3 µm-emitting QCL

B.2.2.1 Effect of varying the Δ value

The device that matched experimental data has a Δ value of 0.11 nm. Table S8 shows a comparison between devices of four different **Δ** values: 0.10 nm, 0.11 nm, 0.12 nm and 0.13 nm. The results show that the η_{sl} value meets our criterion of +/- 4 % variations with respect to the value found to match experiment (i.e., 2.61 W/A for Δ = 0.11 nm), for the Δ range: $0.11\,^{+0.01}_{-0.01}$ nm. More specifically, over that range the η_{sl} value is: $2.61\ \mathrm{W/A}\,^{-2.3\,\%}_{-1.9\,\%}$. Since η_{tr} is basically constant over

Table S8: Changes in η_{sl} with varying the Δ value.

Δ (nm)	J_{th} (kA/cm^2)	J_{max} (kA/cm^2)	η_{sl} (W/A)	η_{tr} (%)	J_{leak}/J_{th} (%)	η_p (%)	η_{sl} x η_p/η_p (W/A)	Error (%)
**0.10**	1.34	5.4	**2.66**	88.8	21.8	**78**	2.65	- **0.2**
**0.11**	1.37	5.42	**2.61**	88.9	23.3	**76.7**		
**0.12**	1.38	5.42	**2.55**	89	25.1	**75**	2.55	**0**
0.13	1.38	5.43	2.47	89	27.4	72.6	2.47	0

the entire range of Δ values, $\eta_{sl} \propto \eta_p$ with negligible error ($\leq$ 0.2 %). The reason for this behavior can be seen from the data in Table S9.

Table S9: Changes in the J_{leak}^{IFR} value with varying the Δ^2 value.

Δ (nm)	J_{leak}^{IFR}/J_{th} (%)	J_{leak}^{IFR} (kA/cm^2)	J_{leak}^{IFR} x Δ^2/Δ^2 (kA/cm^2)	Error (%)
**0.10**	12.3	**0.165**	0.158	- **4.3**
**0.11**	13.93	**0.191**		
**0.12**	15.8	**0.218**	0.227	+ **4.3**
0.13	18	0.248	0.267	+ 7.7

Just as for the other studies, since $\eta_{sl} \propto \eta_p$ one has to look at the behavior of the J_{leak}^{IFR} portion of η_p as a function of the Δ value. As seen from Table S9, the J_{leak}^{IFR} value for the Δ = 0.11 nm case, when scaled with Δ^2 agrees with calculated values with a +/- 4.3 % error. Then, just as for the 4.9 µm-emitting QCL study, the conclusion is the same: the higher the in-plane RMS roughness is, the more IFR-triggered leakage occurs, which explains the decrease in η_{sl} values with increasing Δ value (Table S8).

B3. Summary and Conclusion

The sensitivity analysis to variations in IFR parameters on the performance of the published 4.9 µm- and 8.3 µm-emitting QCLs has revealed that the slope efficiency, η_{sl}, is the most sensitive device characteristic to variations in the L/Δ$_\perp$ ratio and the in Δ values. Maximum errors of +/- 4 % in the η_{sl} value have been chosen as the criterion of a reasonably good fit to experimental data.

For the 4.9 µm-emitting QCLs the acceptable range in L/Δ$_\perp$ values is : $4\,^{+0.5}_{-0.5}$. Over that range the η_{sl} value is: $5.72\ \mathrm{W/A}\,^{+3.8\,\%}_{-2.8\,\%}$. The η_{sl} value increases with increasing L/Δ$_\perp$ value because of less IFR-triggered carrier leakage [7,8].

For the 8.3 µm-emitting QCLs the acceptable range in L/Δ$_\perp$ values is: $4\,^{+1}_{-1}$. Over that range the η_{sl} value is: $2.61\ \mathrm{W/A}\,^{+2.3\,\%}_{-3.1\,\%}$. Just like for the 4.9 µm-emitting QCLs, the η_{sl} value increases with increasing L/Δ$_\perp$ value because of less IFR-triggered carrier leakage.

We don't expect L to be wider than 0.55 nm or narrower than 0.30 nm. Thus, for the $L/\Delta_{\perp}$ value that best fits the experimental data (i.e., 4) the $\Delta_{\perp}$ value may well be in the 0.08-0.14 nm range.

For the 4.9 μm-emitting QCL a comparison of devices of a varying Δ value (0.10/0.13/0.17 nm) vs. devices of constant Δ value (0.13 nm) reveals an ~ 3 % increase in the η_{sl} value of the former over the latter, due to the low-Δ, lattice-matched barrier reducing the IFR-triggered carrier leakage. Varying the Δ value of the moderately strained barrier: $0.13\,^{+0.01}_{-0.01}$ nm, leads to an the η_{sl} value of: $5.72\ \mathrm{W/A}\,^{-3.9\,\%}_{+3.9\,\%}$. The η_{sl} value decreases with increasing Δ value because of increased IFR-triggered carrier leakage [8].

For the 8.3 μm-emitting QCLs the acceptable range in Δ values is: $0.11\,^{+0.01}_{-0.01}$ nm. Over that range the η_{sl} value is: $2.61\ \mathrm{W/A}\,^{-2.3\,\%}_{-1.9\,\%}$. The η_{sl} values decreases with increasing Δ value because of increased IFR-triggered carrier leakage

We conclude that the η_{sl} value variations with varying $L/\Delta_{\perp}$ and Δ values are primarily related to changes in the IFR-triggered carrier leakage. This is an important finding, in that the η_{sl} value is directly related to the maximum CW output power and CW wall-plug efficiency [10]; thus, it becomes a design guideline for device-performance maximization via IFR-scattering engineering as well as via changes in crystal-growth conditions for reducing IFR-triggered carrier leakage.

C. Calculation of the stimulated lifetime for the 4.9 μm-emitting QCL

The analyzed 4.9 μm-emitting QCL [5] was found to have, at threshold, two gain spectra of similar gain peak value. For such a case, calculations of the various lifetimes, as needed to estimate the carrier-leakage current, are not only nontrivial, but cannot take into account nonlinear interactions between different lasing transitions. In turn, we calculated the relative leakage-current density at 1.4 x threshold, where there is only one dominant gain peak for the transition from energy level g_4 and the lower-laser (*ll*) levels. However, at that drive level the laser's output power is 1.14 W; thus, there definitely exists a stimulated field which impacts lifetimes related to the lasing transition. Therefore we calculated the stimulated lifetime, τ_{stim}, which impacts both the lasing-transition lifetime, $\tau_{g4,ll}$, and the global upper-level (*ul*) lifetime, $\tau_{g4,g}$; i.e., the *ul*-level lifetime taking into account transitions to all low-energy states in the active region.

For a standard 3-level laser rate equations [11], the rate of stimulated emission is:

$$Sg_cL_p(n_3 - n_2) \tag{2}$$

where S is defined as the photon flux per period and active-region cross-sectional area, g_c is the gain cross-section [11], L_p is the period length, and n_3 and n_2 are the sheet carrier densities in the *ul* and *ll* levels, respectively. If we want to characterize the rate of stimulated emission in term of a time constant, we write:

$$Sg_cL_p(n_3 - n_2) = (n_3 - n_2)/\tau_{stim} \tag{3}$$

where τ_{stim} is the stimulated lifetime. Thus:

$$\tau_{stim} = 1/\left(Sg_cL_p\right) \tag{4}$$

S can be obtained from the expression for the output power, P_{out} :

$$P_{out} = \frac{hc}{\lambda}(1 - R)(N_pWL_p)S \tag{5}$$

where $hc\ /\ \lambda$ is the photon energy (0.258 eV in this case); N_p is the number of periods (40); R is front-facet power reflectivity (0.11); and W is the buried-ridge width (8 μm). Given L_p = 43.9 nm and P_{out} =1.14 W, S has a value of 2.203x10^{26} photons/sec/cm^2. Using a calculated g_c value of 3.273x10^{-10} cm, from Eq. (4) we obtain τ_{stim} = 3.16 ps.

D. Comparisons to results obtained with extracted abrupt-interfaces IFR parameters
D.1 4.9 μm-emitting QCL

We ran the nextnano NEGF-based model, modified for mid-IR QCLs, with parameters extracted from 4.6 μm-emitting QCLs assumed to have abrupt interfaces [12]: Λ ≅ 9 nm and Δ = 0.12 nm. The obtained L-I and V-I curves are compared to the experimental ones, and the graded-interfaces ones in Fig. S4. The J_{th} value increases from 1.3 kA/cm^2 to 2.2 kA/cm^2, since lasing starts with injection from the 1st-excited injector state, level g_1, at much higher field strength: 84 kV/cm vs. 65.7 kV/cm. The emission wavelength is 4.5 μm, which hints that the laser was designed for 4.6 μm emission. Lasing ceases at a J_{max} value of 3.9 kA/cm^2 where state g_1 reaches resonance with state 4, the *ul* level. Thus, the abrupt-interfaces modeled device behaves like a conventional QCL with pocket injection [13], but no PICT action, in that R_{diff} = 3.2 Ω, and the J_{max} value is basically the same as that of conventional QCLs of same injector-doping level (i.e., ~ 3.8 kA/cm^2).

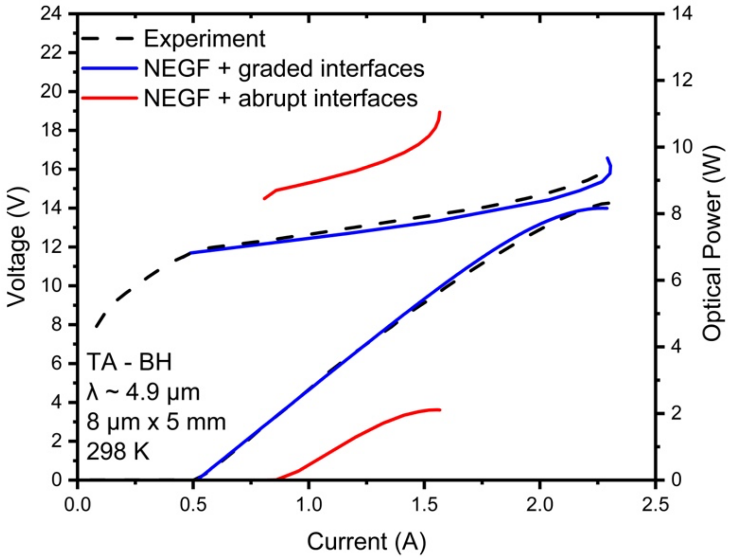

Fig. S4: Comparison of L-I-V curves obtained using IFR parameters extracted assuming abrupt interfaces [11] vs. the experimental L-I-V curves, and the L-I-V curves obtained using graded-interfaces modeling.

As for the maximum wall-plug efficiency it is, as expected, rather low: 8.5 %. Unlike PICT-action QCLs, there is no injection from a prior stage low-energy state into the *ul* level, but just conventional resonant-tunneling injection from a low-energy injector state into the *ul* level. Notably, at resonance there is a large splitting energy (13 meV); that is, strong coupling, but this just goes to show that strong coupling is not enough to achieve PICT action.

D.2 8.3 μm-emitting QCL

We ran the nextnano NEGF-based model, modified for mid-IR QCLs, with parameters extracted from a 8.5 μm-emitting QCLs assumed to have abrupt interfaces [14]: Λ = 9 nm and Δ = 0.10 nm.

The obtained L-I and V-I curves are compared to the experimental ones, and to the graded-interfaces ones in Fig. S5. J_{th} increases from 1.37 kA/cm^2 to 1.98 kA/cm^2, since lasing starts with injection from a state below state 2 of the prior stage (i.e., from state 2') into level g_2, thus at a higher field: 46.6 kV/cm vs. 42.5 kV/cm, compared to injection from state 2 into level 4 in the graded-interfaces case. There is PICT action, but it is significantly weaker than for the graded-interfaces case, as evidenced by a higher R_{diff} value: 1.9 Ω vs. 1.6 Ω, and a lower J_{max} value: 5.2 kA/cm^2 vs. 5.45 kA/cm^2. The slope efficiency is also lower: 2.2 W/A vs. 2.6 W/A, due to higher relative carrier leakage (i.e., 37.6 % vs. 23.3 %). Thus, the maximum wall-plug efficiency: 10 %, is significantly lower than for the graded interfaces case: 17 % [15].

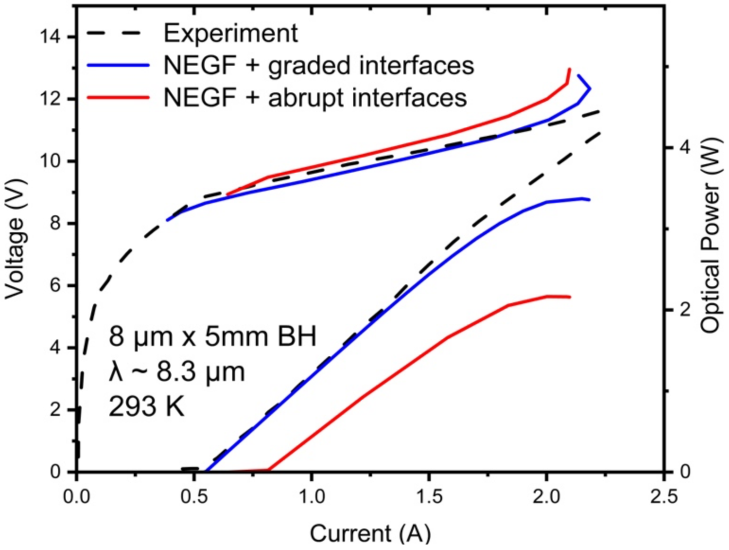

Fig. S5: Comparison of L-I-V curves obtained using IFR parameters extracted assuming abrupt interfaces [13] vs. the experimental L-I-V curves, and the L-I-V curves obtained using graded-interfaces modeling.

References

1. D. Botez, J. C. Shin, J. D. Kirch, C.-C. Chang, L. J. Mawst, T. Earles, "Multidimensional conduction-band engineering for maximizing the continuous-wave (CW) wallplug efficiencies of mid-infrared quantum cascade lasers", *IEEE J. Sel. Top. Quantum Electron.*, vol. 19, no. 4, 1200312, 2013; Correction: *IEEE J. Sel. Top. Quantum Electron.*, vol. 19, no. 4, 9700101, 2013.
2. B. B. Knipfer, S. Xu, J. D. Kirch, D. Botez and L. J. Mawst, "Analysis of interface roughness in strained InGaAs/AlInAs quantum cascade laser structures (λ ~ 4.6 μm) by atom probe tomography," *J. Cryst. Growth*, vol. 583, 126531, 2022.
3. A. Lyakh, R. Maulini, A. Tsekoun, R. Go, C. Pflugl, L. Diehl, Q. J. Wang, F. Capasso, and C. K. N. Patel, "3 W continuous wave room temperature single-facet emission from quantum cascade lasers based on nonresonant extraction design approach," *Appl. Phys. Lett.*, vol. 95, no. 14, 141113, 2009.
4. D. Botez, J. D. Kirch, C. Boyle, K. M. Oresick, C. Sigler, H. Kim, B. B. Knipfer, J. H. Ryu, D. Lindberg III, T. Earles, L. J. Mawst, and Y. V. Flores, "High-efficiency, high-power mid-infrared quantum cascade lasers," *Opt. Mater. Express*, vol. 8, no. 5, pp.1378-1398, 2018; Erratum: vol. 11, no. 7, p.1970, 2021.
5. Y. Bai, N. Bandyopadhyay, S. Tsao, S. Slivken, and M. Razeghi, "Room temperature quantum cascade lasers with 27% wall plug efficiency," *Appl. Phys. Lett.*, vol. 98, no. 18, 181102, 2011.

6. D. Botez, C. -C. Chang, L. J. Mawst, "Temperature sensitivity of the electro-optical characteristics for mid-infrared (λ = 3-16 μm)-emitting quantum cascade lasers," *J. Phys. D: Appl. Phys*. vol. 49, no. 4, 043001, 2016.
7. T. Grange, S. Mukherjee, G. Capellini, M. Montanar, L. Persichetti , L. Di Gaspare, S. Birner , A. Attiaoui, O. Moutanabbir, M. Virgilio, and M. De Seta, "Atomic-scale insights into semiconductor heterostructures: from experimental three-dimensional analysis of the interface to a generalized theory of interfacial roughness scattering," *Phys. Rev. Applied*, vol. 13, no 4, 044062, 2020.
8. C. Boyle, C., K. M. Oresick, J. D. Kirch, Y. V. Flores, L. J. Mawst, and D. Botez, "Carrier leakage via interface-roughness scattering bridges gap between theoretical and experimental internal efficiencies of quantum cascade lasers," *Appl. Phys. Lett*., vol. 117, no. 5, 051101, 2020; Erratum: *Appl. Phys. Lett*., vol.117, no. 10, 109901, 2020.
9. Y.V. Flores, S. S, Kurlov, M. Elagin, M P. Semtsiv and W.T. Masselink, "Leakage current in quantum-cascade lasers through interface roughness scattering," *Appl. Phys. Lett*., vol. 103, no. 16, 161102, 2013.
10. D. Botez and L. J. Mawst, "State-of-the-Art Mid-Infrared QCLs: Elastic Scattering, High CW Power and Coherent-Power Scaling", in *Mid-Infrared and Terahertz Quantum Cascade Lasers*, D. Botez and M. A. Belkin Eds., Cambridge, England: Cambridge University Press, 2023, pp. 72 -77.
11. J. Faist, *Quantum Cascade Lasers,* Oxford, England: Oxford University Press, 2013.
12. A. Bismuto, R. Terazzi, M. Beck, and J. Faist, "Influence of the growth temperature on the performances of strain-balanced quantum cascade lasers," *Appl. Phys. Lett*., vol. 98, no. 9, 091105, 2011.
13. A. Bismuto, R. Terazzi, B. Hinkov, M. Beck, and J. Faist, "Fully automatized quantum cascade laser design by genetic optimization," *Appl. Phys. Lett*., vol. 101, no 2, 021103, 2012.
14. M. Lindskog, J. M. Wolf, V. Trinite, V. Liverini, J. Faist, G. Maisons, M. Carras, R. Aidam, R. Ostendorf , A. Wacker, "Comparative analysis of quantum cascade laser modeling based on density matrices and non-equilibrium Green's functions," *Appl. Phys. Lett*., vol. 105, no.10, 103106, 2014.
15. W. Zhou, Q.-Y. Lu, D.-H. Wu, S. Slivken and M Razeghi, "High-power, continuous-wave, phase-locked quantum cascade laser arrays emitting at 8 μm," *Opt. Express*, vol. 27, no.11, pp. 15776-15785, 2019.
